# The Conoid Associated Motor MyoH Is Indispensable for *Toxoplasma gondii* Entry and Exit from Host Cells

**DOI:** 10.1371/journal.ppat.1005388

**Published:** 2016-01-13

**Authors:** Arnault Graindorge, Karine Frénal, Damien Jacot, Julien Salamun, Jean Baptiste Marq, Dominique Soldati-Favre

**Affiliations:** Department of Microbiology and Molecular Medicine, Faculty of Medicine, University of Geneva, Geneva, Switzerland; Boston College, UNITED STATES

## Abstract

Many members of the phylum of Apicomplexa have adopted an obligate intracellular life style and critically depend on active invasion and egress from the infected cells to complete their lytic cycle. *Toxoplasma gondii* belongs to the coccidian subgroup of the Apicomplexa, and as such, the invasive tachyzoite contains an organelle termed the conoid at its extreme apex. This motile organelle consists of a unique polymer of tubulin fibres and protrudes in both gliding and invading parasites. The class XIV myosin A, which is conserved across the Apicomplexa phylum, is known to critically contribute to motility, invasion and egress from infected cells. The MyoA-glideosome is anchored to the inner membrane complex (IMC) and is assumed to translocate the components of the circular junction secreted by the micronemes and rhoptries, to the rear of the parasite. Here we comprehensively characterise the class XIV myosin H (MyoH) and its associated light chains. We show that the 3 alpha-tubulin suppressor domains, located in MyoH tail, are necessary to anchor this motor to the conoid. Despite the presence of an intact MyoA-glideosome, conditional disruption of *TgMyoH* severely compromises parasite motility, invasion and egress from infected cells. We demonstrate that MyoH is necessary for the translocation of the circular junction from the tip of the parasite, where secretory organelles exocytosis occurs, to the apical position where the IMC starts. This study attributes for the first time a direct function of the conoid in motility and invasion, and establishes the indispensable role of MyoH in initiating the first step of motility along this unique organelle, which is subsequently relayed by MyoA to enact effective gliding and invasion.

## Author Summary

The Apicomplexa phylum groups important pathogens that infect humans and animals. Host cell invasion and egress from infected cells are key events in the lytic cycle of these obligate intracellular parasites. Host cell entry is powered by gliding motility and initiated by the discharge of apical secretory organelles at the site of contact with the host cell. Anchored to the parasite pellicle, the glideosome composed of myosin A and the gliding associated proteins is the molecular machine which translocates the secreted adhesins from the apical to the posterior pole of the parasite and hence propels the parasite into the host cell. *Toxoplasma gondii* exhibits a helical form of gliding motility and as member of the coccidian-subgroup of Apicomplexa possesses an apical organelle called the conoid, which protrudes during invasion and egress and consists in helically organized polymer of tubulin fibers. We have deciphered here the function of a novel myosin associated to the microtubules composing the conoid. Myosin H is essential and prerequisite for motility, invasion and egress from infected cells. This unusual motor links actin- and tubulin-based cytoskeletons and uncovers a direct role of the conoid in motility and invasion.

## Introduction

The phylum of Apicomplexa includes numerous human and animal pathogens that have adopted an obligate intracellular life style and consequently critically depend on active invasion and egress from the infected cells to ensure survival and propagation. Host cell entry is initiated by the attachment and reorientation of the polarized parasites, such that the apical secretory organelles (micronemes and rhoptries) sequentially discharge their contents at the point of contact with the host cell plasma membrane. Both host cell entry and exit are driven by gliding motility, a process involving conserved machinery termed the glideosome, located in the space between the inner membrane complex (IMC) and the parasite plasma membrane where adhesins are translocated from the apical to the posterior pole [1]. Adhesins and other proteins are secreted apically by the micronemes and among them, AMA1 forms a complex with a set of rhoptry neck proteins (RONs) to establish a tight apposition between the parasite and the host cell membrane [2]. This attachment zone forms a ring-like structure called the moving junction [3] and through which the parasite enters the host cell [4]. It is referred here to the circular junction (CJ).

*Toxoplasma gondii* is among the most successful invaders with a third of the world’s human population chronically infected, as well as a broad range of warm-blooded animals. This parasite is responsible for toxoplasmosis, which can lead to severe neurological defects in situations of immunosuppression and in case of congenital infection [5]. The gliding motility of *T*. *gondii* tachyzoites has been experimentally dissected and deconstructed into three forms of motion that include circular and helical gliding and stationary twirling [6]. This has recently been further and more accurately assessed through use of a 3-dimensional, matrigel-based motility assay [7].

As a member of the coccidian subgroup of the Apicomplexa, *T*. *gondii* harbours an additional apical motile organelle termed the conoid, which is connected to two preconoidal rings (PCR) at the top and an apical polar ring (APR) at the bottom that serves as a polarized microtubule organizing centre forming all together the apical complex [8]. This organelle protrudes during gliding, invasion and egress and is formed by a unique polymer of tubulin fibers arranged as a set of counter-clockwise spiralling filaments, ultimately creating a cone-shaped structure [9]. A similar structure is present in other members of the group of Alveolata [10] and has also been recently studied in the Gregarine genus [11]. In *T*. *gondii*, twenty-two subpellicular microtubules (MTs) emerge from the APR, extend in a helical fashion over two thirds of the parasite length and contribute to the overall shape, rigidity and polarity of the parasite [9, 12]. More than 170 proteins have been identified in the conoid enriched fraction including dynein, calcium binding proteins, MORN-domain-containing proteins, centrin, and also some myosin heavy and light chains [8]. More specifically, two proteins, the lysine methyltransferase (AKMT) and the ring protein RNG2 have been localised to the apical complex and functionally associated to parasite motility [13, 14].

The conoid is the first organelle generated during daughter cell scaffold formation [12, 15] and its extrusion was thought to contribute to motility and invasion [16]. Intriguingly, a screen for small molecules inhibiting invasion identified Inhibitor 6 as a compound able to block conoid protrusion without affecting parasite motility [17]. Protrusion is induced by the calcium ionophore A23187 or ethanol, and inhibited by Ca^2+^ chelation (BAPTA-AM), suggesting that Ca^2+^ release from internal stores may act as a key signal to trigger this movement [16, 18]. Additionally sensitivity to cytochalasin D (CD) and to the myosin ATPase inhibitor 2,3-butanedione monoxime (BDM) implicates a role for a myosin motor in conoid protrusion [18].

Micronemes and rhoptries presumably secrete their contents at the tip of the apex. To discharge and inject their contents into host cells, the neck of the rhoptries traverses the apical complex and reaches a point of contact at the tip of the parasite referred to as the porosome [19]. It is unclear whether full conoid protrusion is necessary for rhoptry discharge, in contrast it does not appear to be a prerequisite for the release of microneme content [17].

*T*. *gondii* possesses eleven unconventional myosin heavy chains, many of which are yet to be assigned a role [20]. MyoA is highly conserved across the phylum of Apicomplexa and plays a central role in powering motility [21]. As part of the glideosome, MyoA is anchored to the IMC via its interaction with the Gliding Associated Protein 70 (GAP70) at the level of the apical cap, and with GAP45 throughout the remaining length of the parasite. An additional glideosome based on the class XIV myosin C (MyoC) is restricted to the posterior pole of the parasite and interacts with GAP80 and IMC-associated protein 1 (IAP1) [22]. Although recent work demonstrated that *myoA* gene is dispensable for tachyzoite survival [23], MyoC was later shown to compensate for the absence MyoA and deletion of the two myosin genes was not tolerated [24, 25].

The essential nature of these two myosins during gliding and invasion, points to an important role for actin during these events. Conditional excision of the unique actin gene in *T*. *gondii* (ACT1) resulted in a severe but not complete block in invasion. This led to the postulation of an alternative model for invasion [24, 26]. Despite this, an additional in depth investigation of ACT1 depletion consolidated the importance of actin during invasion [27].

Here we have characterized *T*. *gondii* myosin H (MyoH, XP_002366825), an indispensable actin-based motor associated to the microtubules of the conoid. The tail region of MyoH possesses alpha-tubulin suppressor 1 domains (ATS1) that are a prerequisite for the anchoring of this motor to the conoid. Despite the presence of an intact MyoA-glideosome, depletion of MyoH dramatically impacts on motility, invasion and egress from the infected cells. Importantly, microneme secretion and conoid protrusion remain unaffected in the absence of MyoH. These results highlight a key contribution of the conoid with MyoH acting presumably as a prerequisite initiator of motility at the parasite tip, which is then relayed by MyoA at the level of the IMC.

## Results

### MyoH localizes to the conoid and is critical for parasite survival

MyoH belongs to the class XIVc of myosins and is found restricted to the coccidian subgroup of Apicomplexa [20]. This myosin heavy chain of 1513 amino acids is composed of a head, which maintains ATPase and actin-binding activities, linked via the neck to the tail domain. The unusually large neck domain comprises 8 IQ motifs that presumably serve as binding sites for myosin light chains (MLCs) in order to fulfil structural and regulatory functions. Lastly, the tail domain includes 3 α-tubulin suppressor 1 (ATS1) or RCC1 (Regulator of chromosome condensation 1) domains previously reported to play a role in the regulation of microtubule coordination during the cell cycle of *Saccharomyces cerevisiae* [28] (S1 Fig).

**Fig 1:**
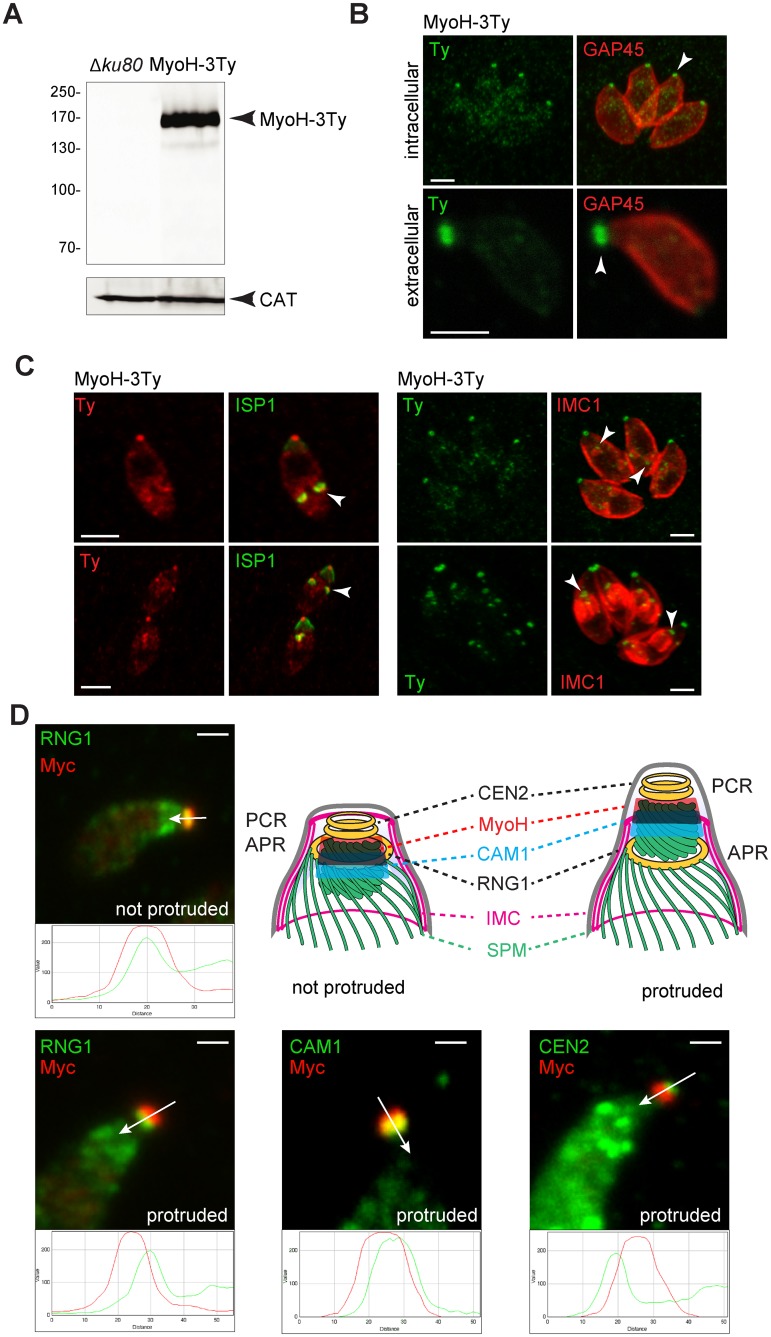
*T*. *gondii* myosin H appears during IMC formation and localizes close to the pre-conoidal rings. (A) TgMyoH-3Ty is found at the predicted molecular weight by western blot (174 kDa). *T*. *gondii* catalase (CAT) was used as loading control. (B) MyoH-3Ty localizes at the extremities of the conoid in intra- (upper panel) and extracellular parasites (lower panel) (arrowhead). GAP45 stains the periphery of the parasites. Scale bar 2 μm. (C) MyoH-Ty localization during parasite division using respectively the IMC sub-compartment protein 1 (ISP1) and the inner membrane complex 1 (IMC1) markers. MyoH (arrowhead) appears early during division and accumulates at the apical end of the newly formed parasites. Scale bar 2 μm. (D) The precise localization of MycMyoH relatively to the conoid markers: RING1 (RNG1) at the apical polar ring (APR); calmodulin 1 (CAM1) in the middle part of the conoid and centrin 2 (CEN2) at the pre-conoidal rings (PCR). Co-localizations are assessed by the RGB profile plots determined using ImageJ along the arrows. Conoid protrusion in extracellular parasites was induced with A23187. IMC: inner membrane complex, SPM: subpellicular microtubules. Scale bar 1 μm.

To determine the subcellular localization of MyoH, the gene was modified at the endogenous locus by the insertion of 3 epitope tags (Ty) via single homologous recombination to produce MyoH-3Ty. In accordance with its predicted amino acid sequence, MyoH-3Ty has an apparent molecular weight of 170 kDa by western blot analysis (WB) (Fig 1A). Indirect immunofluorescence assay (IFA) revealed that this motor is localized to the apical pole, at the tip of both intracellular replicating and extracellular motile parasites and is produced early during parasite division when the daughter IMCs began to emerge (Fig 1B and 1C). In addition, the same localization was observed when the endogenous locus was modified to introduce a myc tag at the N-terminus of MyoH under the control of a tetracycline(tet)-regulatable promoter, without impacting on targeting and function.

Based on a series of IFAs with markers of the tachyzoite apex, we concluded that MyoH is positioned at the tubular core of the conoid. RNG1, a marker of the APR [29], co-localized with myc-MyoH in extracellular parasites but gave a distinct signal upon conoid protrusion (Fig 1D). Partial co-localization was observed between calmodulin 1 (CAM1 [8]) and MyoH (Fig 1D). In contrast, no co-localization was observed between MyoH and centrin 2 (CEN2) which is located at the PCR [8]. Moreover, MyoH-3Ty was shown to form a ring at the top of the parasite by using three-dimensional reconstruction of the MyoH-3Ty staining by super-resolution microscopy (SIM) (S1 Movie). Taken together, these results indicate that the MyoH ring is restricted to the upper part of the conoid, close to the PCR, as depicted in the scheme of Fig 1D.

**Fig 2:**
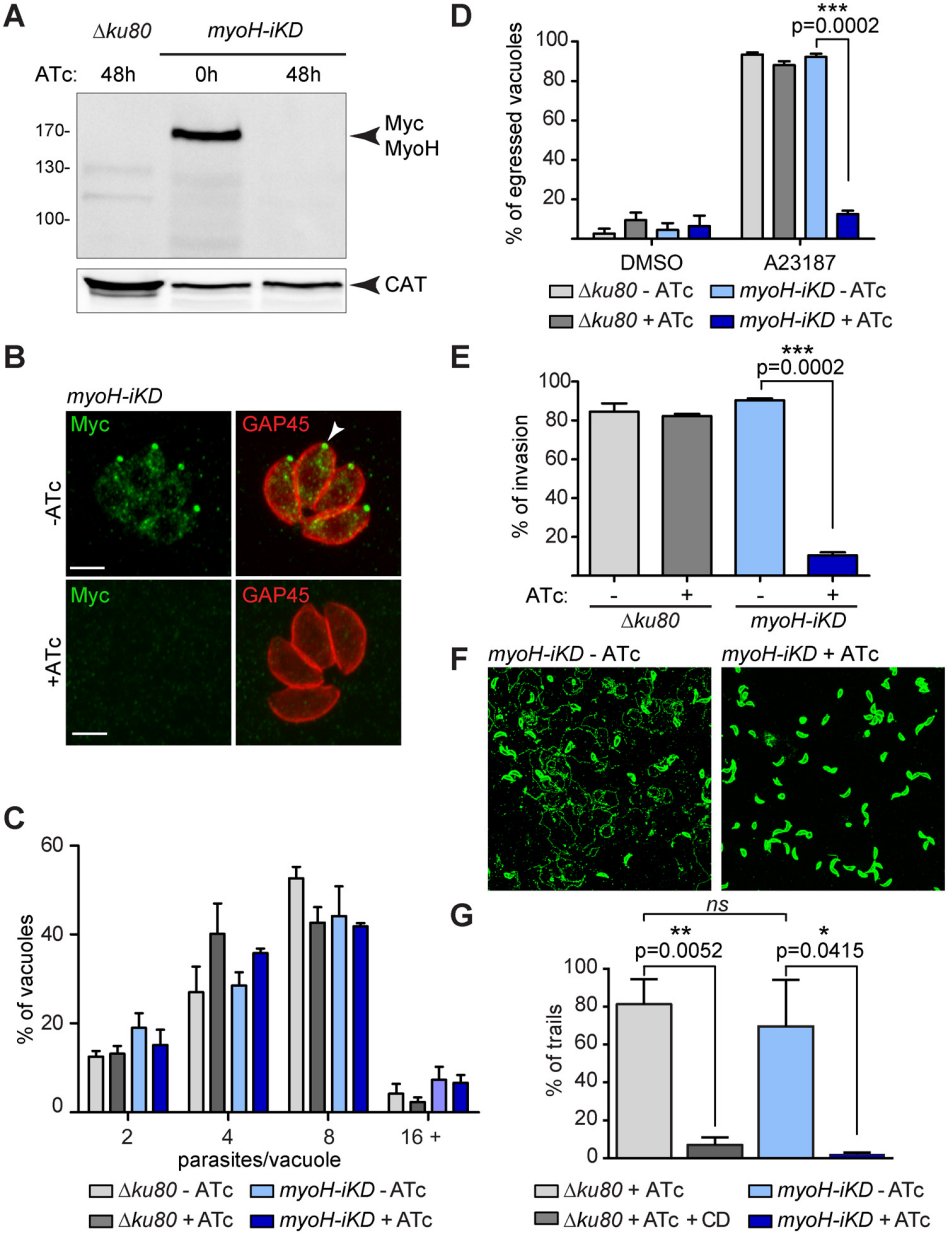
*T*. *gondii* myosin H is involved in gliding motility, invasion and egress. (A) Inducible MyoH (Myc-MyoH) migrates at the predicted molecular weight (171 kDa) by western blot and is down-regulated to undetectable level upon 48 h of ATc treatment. Catalase was used as a loading control. (B) Myc-MyoH localizes at the conoid (arrowhead) and is not detectable after 48 h of ATc treatment. Scale bar 2 μm. (C) Intracellular growth assay performed on *myoH-iKD* and *Δku80* strains upon 48 h incubation ± ATc revealed no altered phenotype. Data are presented as mean ± SD. (D) Calcium ionophore-induced egress assay of *myoH-iKD* and parental (*Δku80*) strains performed by treating the parasites with DMSO or A23187 for 7 min after 48 h ± ATc. Results are expressed as percentage of ruptured vacuoles and represented as mean ± SD. (E) The invasion capacity of *myoH-iKD* and parental (*Δku80*) parasites was evaluated after 48 h ± ATc. Results are expressed as percentage of invading parasites and represented as mean ± SD. (F) Gliding assays performed with *myoH-iKD* strain after 48 h ± ATc. The trails are revealed by indirect fluorescence microscopy using anti-SAG1 antibodies. (G) Quantification of the gliding assay. Results are expressed as percentage of trails detected per field and normalized to the total number of parasites per field. Data are presented as mean ± SD. CD: Cytochalasin D. For (D), (E) and (G), the significance of the results was assessed using a parametric paired t-test and the two-tailed p-values are presented on the graphs, ns: non-significant.

*TgMyoH* was refractory to gene disruption by single homologous recombination in the *RHΔku80* (*Δku80)* strain, suggestive of the crucial nature of this motor. We subsequently undertook conditional disruption approaches and readily obtained an inducible knockdown strain by swapping the endogenous promoter with a tet-repressive promoter (S2A Fig). The resulting strain (*myoH-iKD*) was confirmed by genomic PCR analyses (S2B Fig). WB and IFAs performed on parasites incubated for 48 h in presence of anhydrotetracycline (ATc) revealed a tight regulation of the inducible Myc-MyoH (Fig 2A and 2B).

### MyoH is indispensable for all glideosome-associated functions

**Fig 3:**
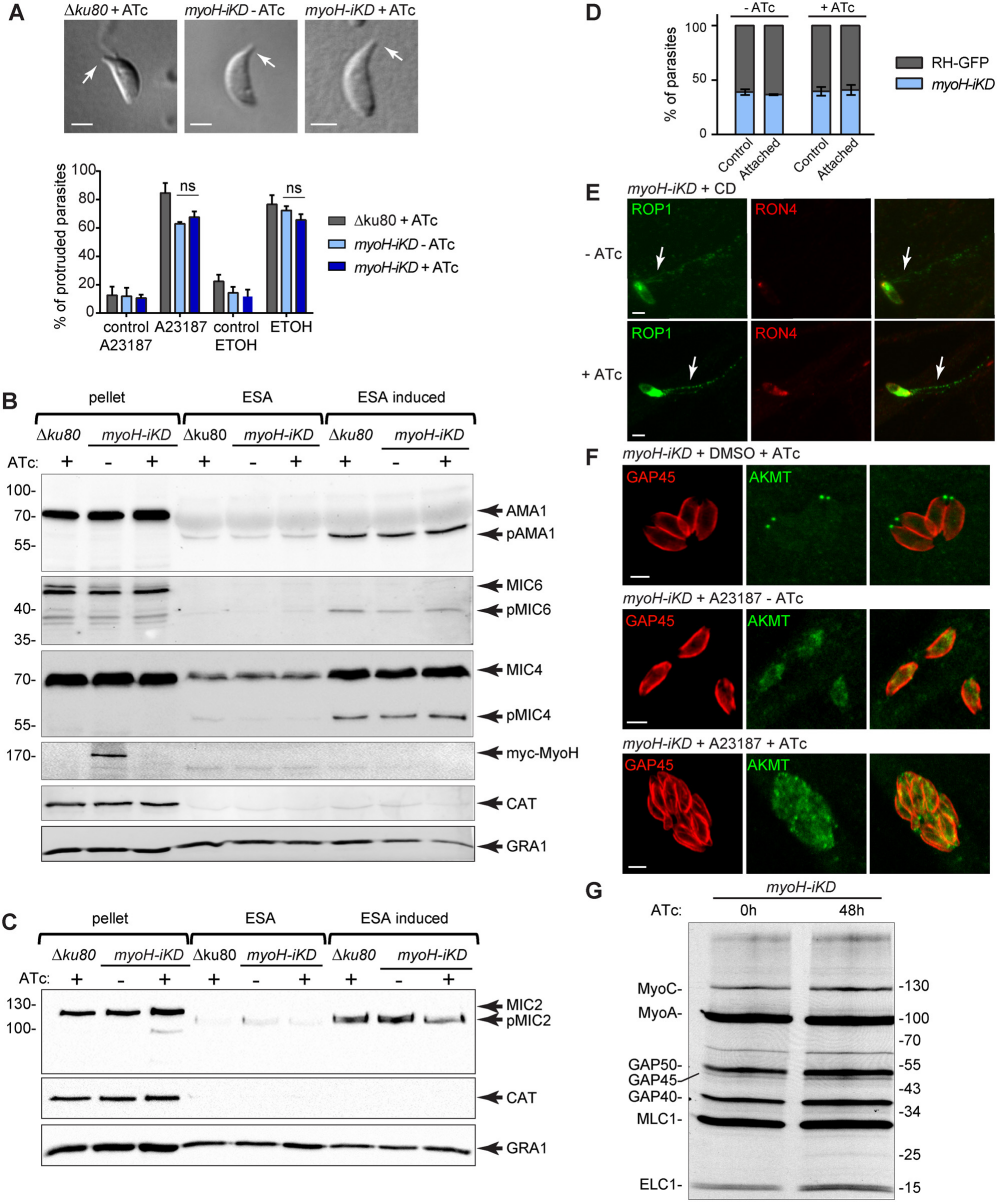
*T*. *gondii* myosin H is not involved in conoid protrusion, microneme or rhoptry secretion, host cell attachment, AKMT relocalization, or glideosome assembly. (A) Conoid protrusion assay performed on wt (*Δku80*) and *myoH-iKO* lines ± ATc for 48 h. DIC pictures are representative of each condition with the protruded conoid indicated by an arrow. Scale bar 1 μm. The graph represents the percentage of conoid protrusion induced by 3 μM of A23187, or 0.5 M ethanol (ETOH) and their respective controls. Data are represented as mean ± SD. The significance of the results was assessed using a parametric paired t-test, ns: non-significant. Microneme secretion assay performed on wt (*Δku80*) and *myoH-iKO* lines ± ATc for 48 h and analyzed by western blot using the following micronemal proteins (MICs); apical membrane antigen 1 AMA1, MIC4, MIC6 (B) or MIC2 (C). “p” correspond to the predicted cleaved forms of the different micronemal proteins analyzed. Catalase (CAT) was used as cytosolic control and dense granule 1 (GRA1) as control for constitutive secretion. ESA: excreted/secreted antigens, ESA-induced: induction with 0.5 M ethanol. (D) Attachment assay performed with a mixed population of wt (RH-GFP) and *myoH-iKD* ± ATc for 48 h. Controls represent the initial percentage of each line prior to the attachment assay. Data are represented as mean ± SD. (E) E-vacuole assay performed on the *myoH-iKD* line treated ± ATc for 48 h. CD: cytochalasin D. Scale bar 2 μm. (F) Cytosolic re-localization of the apical complex lysine methyltransferase (AKMT) upon induction with 3 μM of A23187 in presence or absence of MyoH. Scale bar 2 μm. (G) Co-IP experiments performed with anti-GAP45 antibodies on *myoH-iKD* parasites treated for 48 h ± ATc and metabolically labeled with [S^35^]-methionine/cysteine.

To explore the phenotypic consequences of MyoH depletion, plaque assays were performed with *myoH-iKD* parasites in the presence or absence of ATc for seven days. The absence of detectable plaques upon ATc treatment was indicative of a severe defect in one or more steps of the lytic cycle (S2C Fig). To pinpoint the step(s) affected by MyoH depletion, a series of targeted assays were performed. Parasite replication was unaltered, as scored by intracellular growth assay (Fig 2C). The biogenesis and positioning of the secretory organelles implicated in motility and/or invasion were not affected (S2D Fig). In contrast, egress induced by addition of the calcium ionophore A23187 and host cell invasion were severely impaired in the presence of ATc when compared with untreated parasites falling to 12% and 10%, respectively (Fig 2D and 2E, S2 and S3 Movies). Since invasion and egress critically depend on parasite motility, gliding assays were performed using *myoH*-*iKD* and uncovered a dramatic defect (Fig 2F). Assessment of the number of trails revealed a phenotype as severe as treatment with CD which was previously demonstrated to abrogate gliding motility [30] (Fig 2G). To determine if MyoH had an impact on conoid protrusion, both the reversible ethanol inducer [18] and the stronger A23187 inducer [31] were compared. Despite the suggestive localization of MyoH, its depletion revealed no significant reduction of conoid protrusion upon either stimulation (Fig 3A).

The essential nature of MyoH gene and the contribution of this motor in invasion and egress was confirmed by a parallel strategy leading to the transient excision of the 3’UTR of the gene and the concomitant destabilization of the residual transcript as previously described [32]. To this aim the endogenous locus of *MyoH 3’UTR* was replaced by an excisable 3’UTR followed by U1 sequences to generate MyoH-3Ty-floxU1 strain which was readily obtained. As anticipated, transient transfection of these parasites with a Cre-GFP expressing vector led to the disappearance of MyoH by IFA (S2E Fig). Concordantly, the parasites expressing transiently Cre-GFP (lacking MyoH expression) were significantly impaired in egress and invasion (S2F and S2G Fig).

### MyoH is not necessary for microneme and rhoptry discharge

Depletion of MyoH recapitulates the same phenotypic consequences as in the previously described mutants affecting microneme discharge [17, 33]. Consequently, MyoH depleted parasites were carefully scrutinized for microneme proteins secretion. Subsequent assays revealed that upon ethanol stimulation, the microneme proteins AMA1, MIC6 and MIC4 were normally secreted in the absence of MyoH as indicated by their presence in the excretory-secretory antigen (ESA) fraction of *myoH-iKD* parasites (Fig 3B). The same results were obtained with MIC2 (Fig 3C) whereas its secretion was completely blocked when parasites were treated with compound 2 (S2H Fig). Like compound 1, compound 2 inhibits cGMP-dependent protein kinase, which critically contributes to the signalling cascade leading to microneme secretion [34, 35]. Discharge of MIC2 at the parasite surface did not accumulate at the tip in absence of MyoH (S2I Fig). Concordant with an unaltered microneme secretion, host cell attachment performed on MyoH depleted parasites was not affected when compared with GFP expressing wild type parasites (Fig 3D).

The impact of MyoH depletion was also tested in a rhoptry secretion assay whereby formation of empty vacuoles (e-vacuoles) in the presence of CD was monitored as an indicator of rhoptry discharge [36]. CD pre-treated *myoH-iKD* parasites +/- ATc showed no defect in e-vacuole formation (Fig 3E), indicating that rhoptry secretion is not affected in *myoH-iKD* parasites.

### MyoH acts downstream of AKMT and does not contribute to MyoA-glideosome formation

Members of the Apicomplexa possess a unique AKMT at the conoid that rapidly re-localizes in the presence of egress-stimulating signals in tachyzoites [14]. Interestingly, *akmt-KO* parasites share with *myoH-iKD* the same severe defect in invasion, egress and gliding motility while the substrate(s) and function of this intriguing enzyme are not known [14, 37]. To determine if MyoH function is linked to that of AKMT, we investigated the A23187-dependent cytoplasmic redistribution of AKMT in the absence of MyoH. AKMT re-localization was not affected in *myoH-iKD* parasites treated ± ATc (Fig 3F) however some residual AKMT protein could still be observed in the A23187-induced parasites depleted for MyoH. This suggests either an independent role for MyoH in gliding, or a role likely downstream of the AKMT signalling cascade.

Finally, the assembly and composition of the MyoA-glideosome was examined to ensure that depletion of MyoH did not indirectly impact on glideosome functioning. Both GAP45 and MLC1 were distributed evenly along the IMC in *myoH-iKD* ± ATc parasites (S2D and S2J Fig). Immunoprecipitation of the MyoA-glideosome revealed no alteration of its composition in the absence of MyoH (Fig 3G), however we cannot entirely rule out that this motor would fail to be activated in the absence of MyoH.

### Parasites lacking MyoH have lost virulence and the ability to induce seroconversion in mice

Parasites impaired in gliding motility such the *myoA-iKD* strain loose virulence in the mouse model [21]. Similarly, animals infected with *myoH-iKD* and supplemented with ATc in their drinking water survived infection whereas in the absence of ATc the mice succumbed to infection 8 days post-inoculation (S3A Fig). However, and in contrast to MyoA depletion, infection with parasites depleted in MyoH led to a more radical phenotype *in vivo* since the surviving mice failed to seroconvert after 20 days (S3B Fig) and concordantly showed no protection against a subsequent challenge.

### MyoH is associated with the microtubules of the conoid via the neck and tail domains

**Fig 4:**
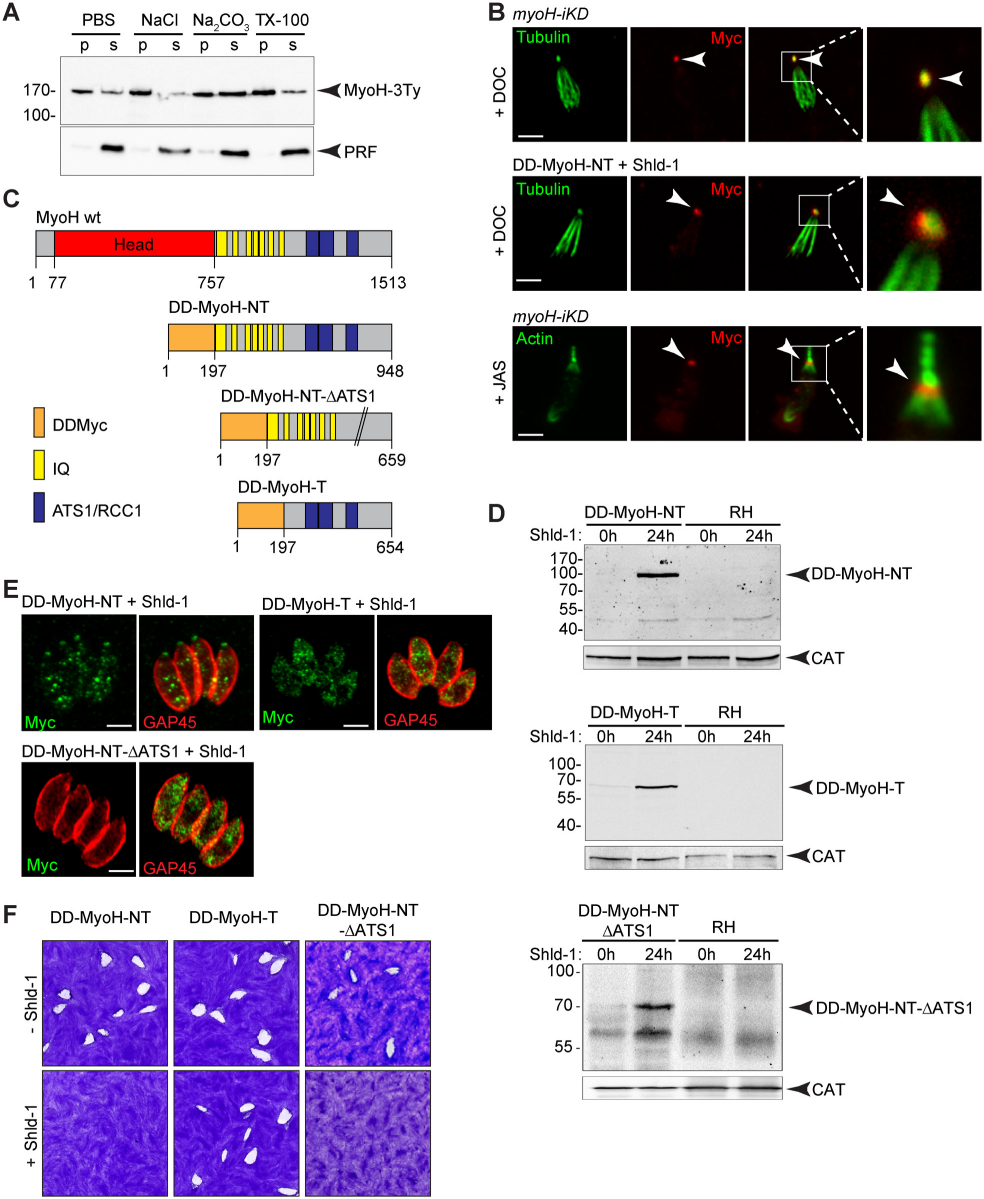
The ATS1 domains of *T*. *gondii* myosin H are necessary for its conoid localization. (A) MyoH-3Ty solubility was evaluated by fractionation after extraction in PBS, PBS/NaCl, PBS/Na2CO3 or PBS/Triton X-100. Their distribution in different fractions was assessed by western blot using anti-Ty antibodies. The profilin (PRF) was used as soluble control. (B) Deoxycholate (DOC) extraction shows that MycMyoH (upper panels) and DD-MyoH-NT-ΔATS1 (middle panels) remain bound to the conoid (arrowhead). Actin polymerization using jasplakinolide at 1 μM (JAS) showed MycMyoH strongly associated to the conoid (arrowhead) and not to the actin-containing apical extensions (lower panels). Scale bar 2 μm. (C) Schematic representation of the TgMyoH and FKBP destabilization domain (DD) constructs. MyoH contains eight IQ motifs and three ATS1/RCC1 domains. NT: Neck and Tail, T: Tail. (D) Western blot analysis using anti-Myc antibodies shows stabilization of DD-MyoH-NT (100 kDa), DD-MyoH-T (64 kDa), and DD-MyoH-NT-ΔATS1 (76 kDa) after 24 h of Shield-1 (Shld-1) treatment. Catalase (CAT) serves as loading control. (E) Only DD-MyoH-NT is present at the conoid. (F) Plaque assays performed with DD-MyoH-NT, DD-MyoH-T, and DD-MyoH-NT-ΔATS1 were fixed after 7 days. A strong impairment in the lytic cycle was observed for DD-MyoH-NT and DD-MyoH-NT-ΔATS1 in the presence of Shld-1.

To glean further insight into MyoH functioning, we determined its solubility using detergent partitioning and carbonate extraction (Fig 4A). A large portion of MyoH is insoluble and likely tightly associated with the parasite cytoskeleton. Treatment of extracellular parasites with deoxycholate (DOC) prior to IFA preserves the association of MyoH with the intact MTs of the conoid (Fig 4B). Since myosins traditionally bind to actin filaments, we artificially induced apical actin polymerization using jasplakinolide (JAS). This assay revealed that MyoH remained strongly anchored to the conoid, again attesting for the robust direct or indirect interaction with MTs (Fig 4B). To determine which part of MyoH interacts with the MTs, we generated transgenic parasites in which a Shield-regulated destabilisation domain (DD, [38]) was appended to extra-copies of truncated forms of MyoH (Fig 4C and 4D). Given the absence of a coiled-coil domain, MyoH is not predicted to operate as a dimer and hence expression of the neck and tail region (DD-MyoH-NT), neck and tail region without ATS1 domains (DD-MyoH-NT-ΔATS1) or tail domain only (DD-MyoH-T) were not anticipated to interact with endogenous full length MyoH. However, overexpression of DD-MyoH-NT, which localized to the conoid, exhibited a strong dominant negative effect (Fig 4F). In sharp contrast, DD-MyoH-T remained cytosolic and its expression was neutral for parasite survival (Fig 4E and 4F). Insertion of a GFP in DD-MyoH-NT (DD-Myc-GFP-MyoH-NT) did not perturb the localization or the expression of the truncated MyoH (S3C and S3D Fig). Metabolic labelling followed by co-IPs of DD-Myc-GFP-MyoH-NT showed no band detected at the wt size of MyoH (170 kDa) suggesting that no deleterious heterodimer is formed with endogenous MyoH (S3E Fig). Since the phenotype of DD-MyoH-NT overexpression perturbs organelle biogenesis (S3F Fig) these results led us to conclude that the numerous IQ motifs could titrate out MLC(s) shared by other myosin motors, compromising their function and hence explaining the toxic effects observed. Concordantly, DD-MyoH-NT-ΔATS1 failed to localize to the conoid but still exhibited a dramatic effect on parasite growth again likely attributable to the over-expression of the neck domain (Fig 4E and 4F). Taken together these results suggest that the ATS1 domains are either directly involved in the association of MyoH with the conoid MTs or indirectly by interacting with conoid specific microtubule-associated proteins (MAPs). Additionally, the neck domain of MyoH appears to participate in bringing the motor to its site of action.

### *T*. *gondii* MyoH motor is associated with at least three distinct myosin light chains

**Fig 5:**
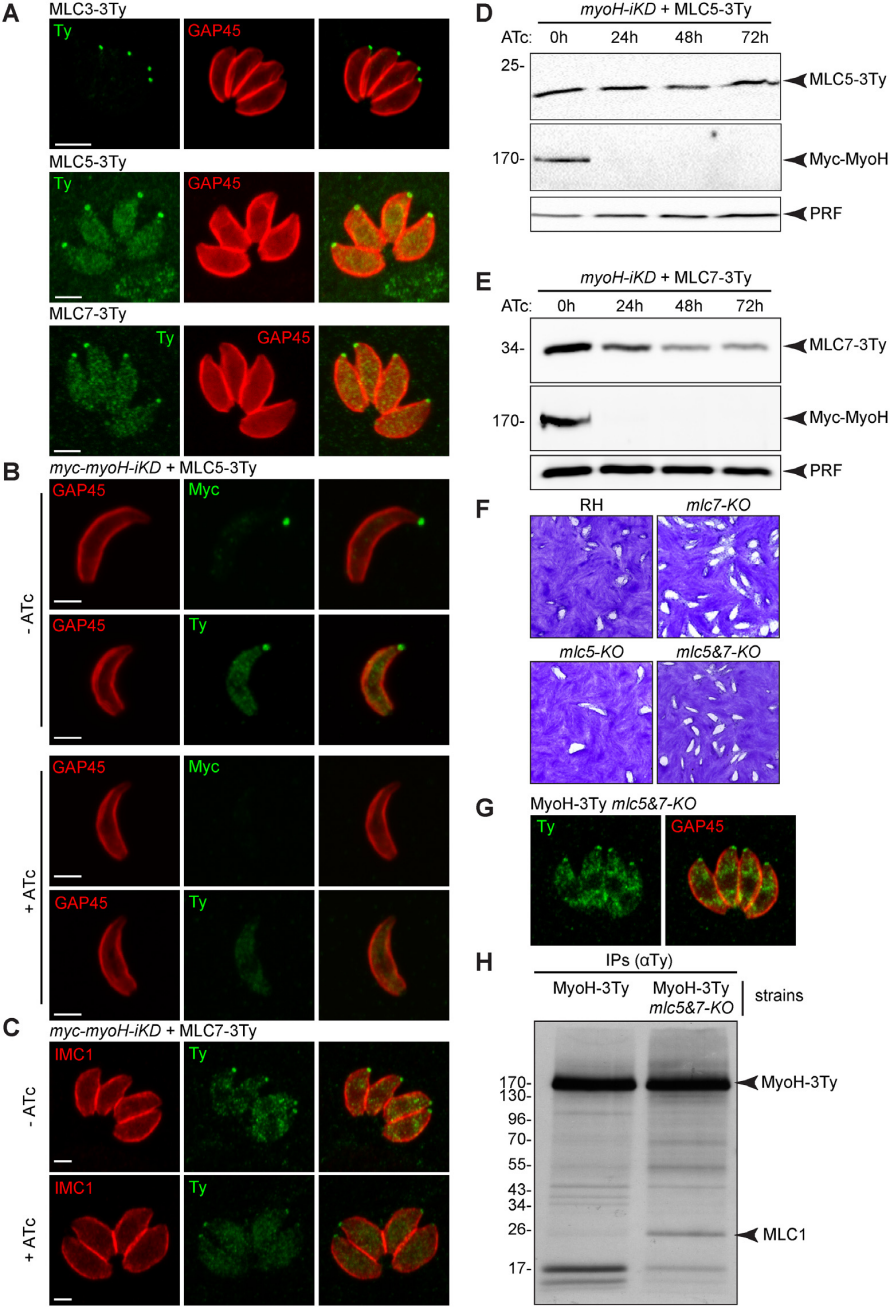
*T*. *gondii* myosin H is associated with three myosin light chains. (A) Endogenously tagged myosin light chains (MLC) 3, 5 and 7 localized to the conoid. Scale bar 2 μm. (B) MLC5 localization at the conoid is dependent upon the presence of TgMyoH. Scale bar 2 μm. (C) MLC7 localization at the conoid is independent on the presence of TgMyoH. Scale bar 2 μm. (D) No decrease of the MLC5 level was observed by western blot upon depletion of TgMyoH. Profilin (PRF) serves as loading control. (E) A decrease of the MLC7 level was observed by western blot upon depletion of TgMyoH. Profilin (PRF) serves as loading control. (F) Plaque assays performed with RH (parental strain), *mlc5-KO*, *mlc7-KO* and *mlc5&7-KO* lines and fixed after 7 days showed no defect in the lytic cycle. (G) TgMyoH localization at the conoid is independent on the presence of MLC5 and MLC7. (H) Metabolic labeling followed by Co-IP experiments performed with anti-Ty antibodies on MyoH-3Ty and MyoH-3Ty-*mlc5&7-KO* strains. A band corresponding to the size of MLC1 was detected.

MyoH is predicted to possess 8 putative IQ motifs that are presumed to bind to one or more MLC(s). *T*. *gondii* possesses at least seven MLCs that have been previously localized by expression of tagged second copies and only MLC5 (TGME49_311260) was reported to localise to the conoid [39]. However, expression of second copies could lead to localization artefacts due to over or improper timing of expression. The MLCs were therefore epitope-tagged via single homologous recombination at the endogenous locus and in addition to MLC5, MLC3 (S3G Fig) and MLC7 were found to localize exclusively to the conoid (Fig 5A). Immunoprecipitation of MLC5 and MLC7 revealed the co-IP of a protein with a size corresponding to the MyoH (S3H Fig). Due to its insolubility, immunoprecipitation of MLC3 could be not investigated. The identity of MyoH was confirmed by mass spectrometry with 11 and 43 unique peptides corresponding to MyoH in the MLC5 and MLC7 co-IPs, respectively (S3I Fig and S1 Dataset). Given the poor solubility of MyoH, the identification of potentially loosely bound partners interacting with this motor would be hampered due to the stringent co-IP conditions utilised in these assays.

In light of these findings we subsequently Ty-tagged MLC5 and MLC7 at their endogenous locus in the *myoH-iKD* background strain in order to investigate their fate in the absence of MyoH. Upon MyoH depletion, MLC5 became cytosolic (Fig 5B) although its expression level quantified by WB was unchanged even after 72 h of ATc treatment (Fig 5D). Interestingly, upon MyoH depletion, MLC7 was still present at the conoid (Fig 5C) but its level of expression decreased after 24 h and stabilized after 48 h of ATc treatment (Fig 5E). To further investigate the contribution of MLC5 and MLC7 in MyoH function, the corresponding genes were individually and concomitantly deleted using CRISPR/Cas9 [40] to introduce out of frame mutations in RH strain parasites. Gene disruption mutants were confirmed by sequencing of the corresponding locus (S4A Fig).

In the absence of both MLC5 and MLC7, there was no significant loss in parasite fitness as revealed by plaque assay (Fig 5F), indicating that these accessory light chains are dispensable for MyoH localization to the conoid (Fig 5G) and for its function. Metabolic labelling followed by co-IPs using MyoH-3Ty and MyoH-3Ty-*mlc5&7*-KO revealed a band at the size of MLC1 (Fig 5H). This interaction was confirmed by co-IPs using MLC1 antibodies and demonstrated that MyoH interacts with MLC1 not only in *mlc5&7-KO* where MyoH was subsequently endogenously tagged but also in wt situation (S4B Fig). No detectable change in MLC1 distribution at the parasite tip was observed when comparing IFAs performed on MyoH-3Ty and MyoH-3Ty-*mlc5&7*-KO strains (S4C Fig).

### *T*. *gondii* myosin H translocates the circular junction from the parasite tip to the apical cap

**Fig 6:**
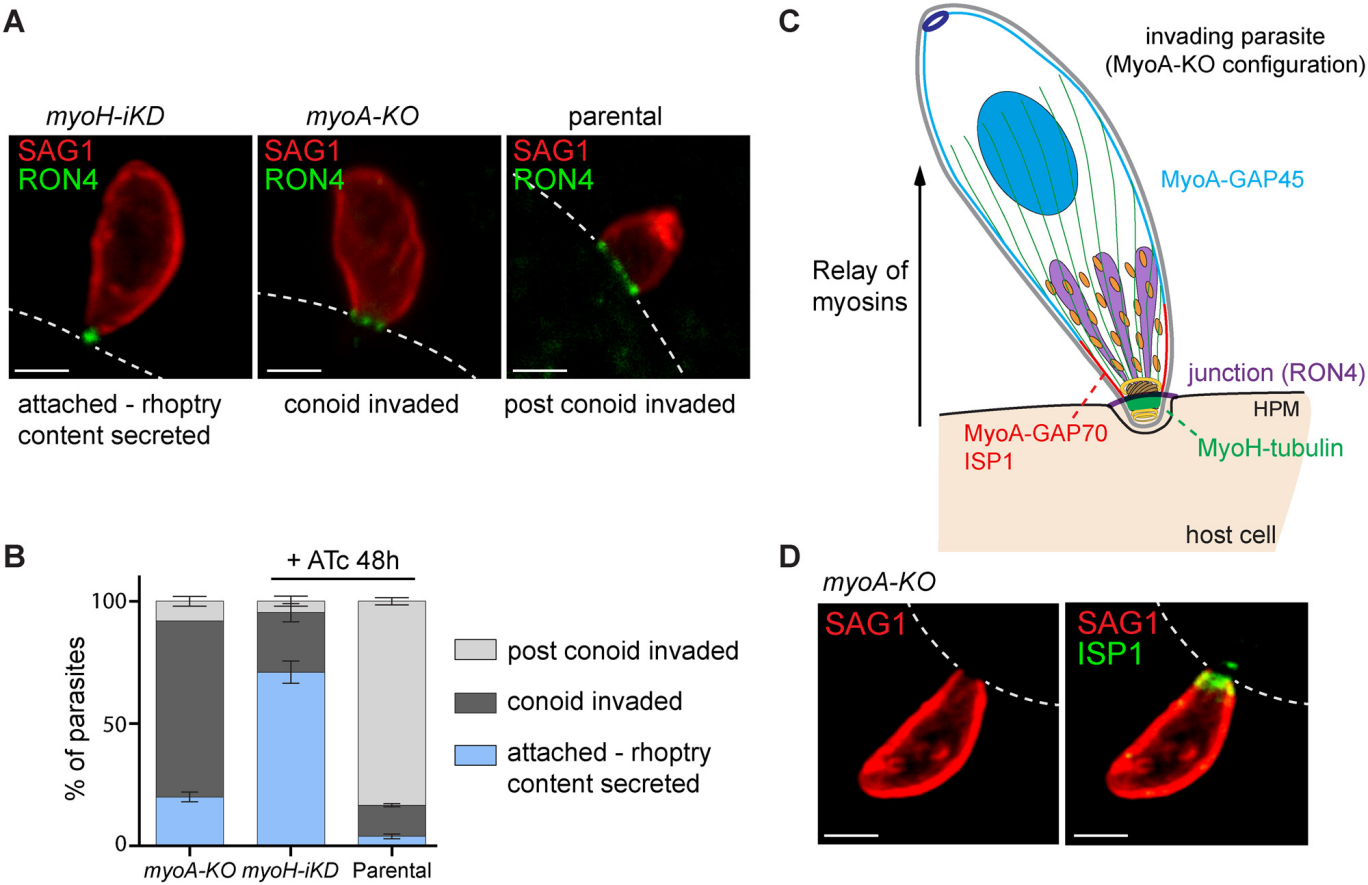
*T*. *gondii* myosin H is part of a conoidal glideosome and triggers the first step of invasion. (A) Invasion pulse of 7 min using *myoH-iKD* + 48 h ATc, *myoA-KO* and a parental strain. The surface antigen 1 (SAG1) and the rhoptry neck secreted protein (RON4) were used to visualize respectively the parasite surface and the circular junction formed by invading parasites. The host cell plasma membrane is illustrated by dashed white lines. Depletion of MyoH blocked most of the parasites at the attachment step, prior to circular junction translocation (RON4 is detectable as a dot). The *myoA-KO* are blocked predominantly during conoid penetration, probably at the IMC level (RON4 is visible as a ring below the conoid). Most of the parental parasites completed penetration but some of them were captured at any step of the entry process (RON4 is detectable as ring at various position along the parasite). Scale bar 1 μm. (B) Quantification of the invasion pulse described in (a). Parasites were scored as attached, conoid invaded up to the IMC or post conoid invaded. Results are expressed as mean ± SD. (C) Schematic representation of the MyoH and MyoA relay at the conoid–IMC interface delineated by ISP1 (IMC sub-compartment protein 1). The scheme represents a parasite in the *myoA*-KO situation as described in (a). HPM: host plasma membrane. (D) Co-localization of SAG1 with the apical cap marker ISP1 after pulse invasion in the *myoA-KO* cell line. Scale bar 2 μm.

To define the step at which MyoH participates in invasion, we performed pulses of invasion and documented the events by IFA using anti-RON4 as a marker of the CJ. In addition and prior to permeabilization with saponin, the accessible extracellular part of the parasites was stained with anti-SAG1 (major surface antigen 1). The *myoH-iKD* parasites under ATc treatment were compared to wild type and *myoA-KO* parasites for their respective abilities to invade host cells within 7 min (Fig 6A). While the majority of wild type parasites successfully completed invasion within this short time, most parasites depleted in MyoH were simply attached to the host cell by their tip with the CJ forming only a dot (Fig 6A and 6B). In sharp contrast, *myoA-KO* parasites successfully inserted their conoid into the host cell, however it must be noted that the CJ is arrested at the beginning of the cap where MyoA is anchored via its interaction with GAP70 [22] (Fig 6C). To confirm that only the tip of the parasite including the conoid entered into the host cell, *myoA-KO* parasites were stained with a marker of the apical cap, anti-ISP1 [41] following pulse invasion (Fig 6D). In *myoA-KO* parasites, SAG1 labelling is interrupted at the level of ISP1 confirming that only the tip of the parasite has penetrated into the host cell.

## Discussion

**Fig 7:**
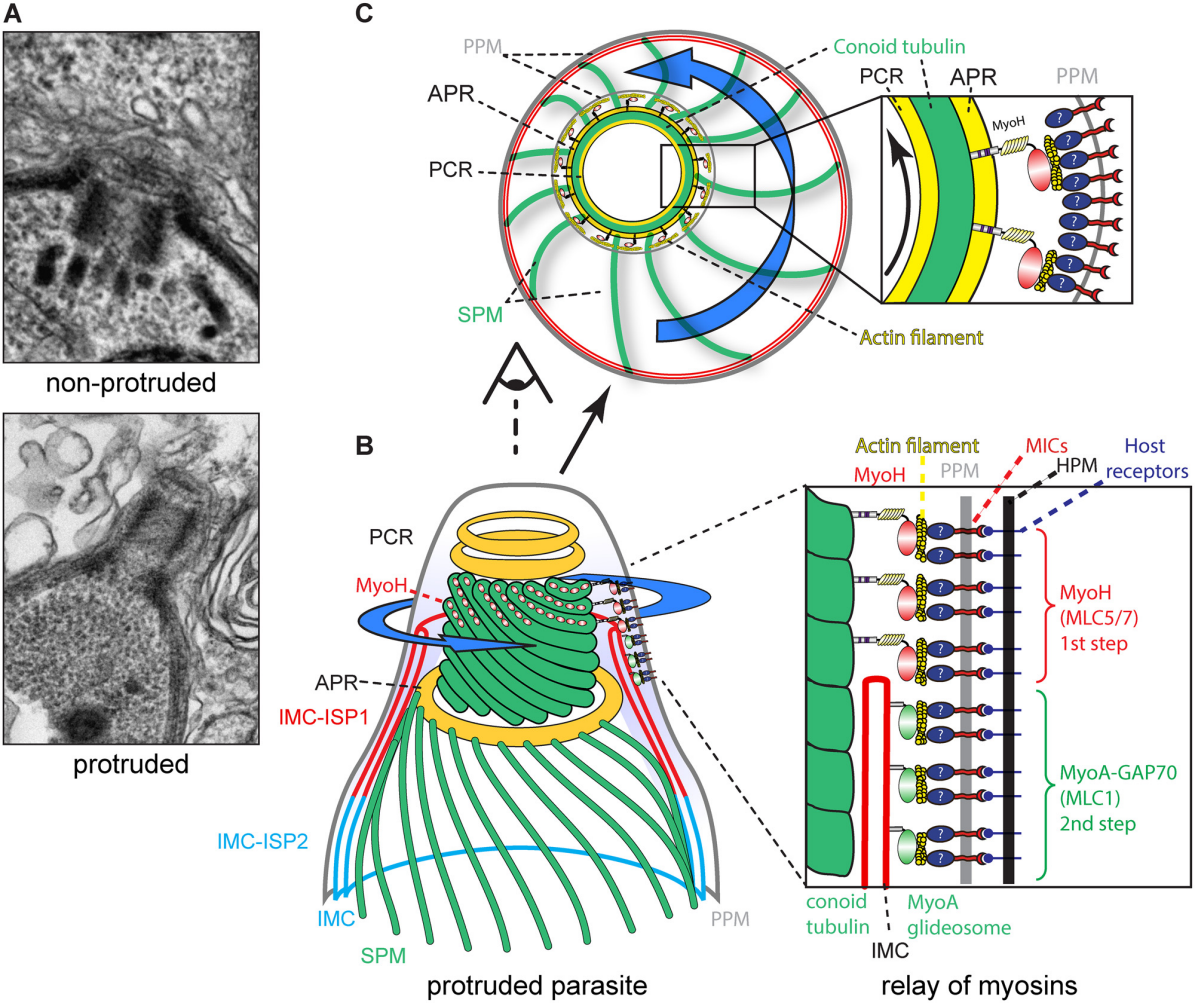
Model. (A) Electron micrographs depicting the conoid in non-protruded and protruded parasites. (B) Schematic representation of the MyoH and MyoA relay at the conoid-IMC interphase. MyoH (red hoops) localized at the upper part of the conoid, close to the pre-conoidal rings (PCR). The translocation of the adhesin complexes by MyoH (blue arrow), probably via actin, are further relayed at the level of the IMC by MyoA-GAP70 in the cap region and then by MyoA-GAP45 along the rest of the parasites (see Fig 6C). APR, apical polar ring; SPM, subpellicular microtubules; PPM, parasite plasma membrane. (C) Upper view of the conoid with MyoH initiating the translocation of the adhesin complexes in a possible corkscrew-like trajectory which is likely relayed at the level of the subpellicular microtubules indirectly connected to the MyoA-glideosome.

The conoid is a mysterious organelle, originally identified as 13 internal microtubule protofilaments of α-tubulin first described in *T*. *gondii* by De Souza in 1974 [9, 42]. Since then, a number of studies have shed light on its structure and its calcium-dependent protrusion/retraction (Fig 7A), however none have assigned any definitive function to this enigmatic and dynamic organelle [8, 18, 43]. Here we demonstrate that MyoH is distributed to the upper part of the conoid, close to the pre-conoidal ring and probably outside of the conoid structure as suggested by the MyoH distribution visualized in the S1 Movie. This myosin of the class XIV is predominantly insoluble in the presence of detergent and remains attached to the conoid upon deoxycholate extraction, strongly indicating a direct or indirect interaction with the MTs constituting this organelle. This association of MyoH to the conoid MTs could involve either the recognition of a specific conformation or posttranslational modification (such as methylation) of the MTs or alternatively implicates some conoid specific MAPs. Expression of the neck and tail regions of MyoH in the presence or absence of the ATS1 domains points toward their involvement in binding MTs either directly or indirectly. With its tail anchored to the conoid, MyoH is presumably facing the plasma membrane in a similar orientation as MyoA, which is anchored via MLC1 and GAP70 to the apical cap, and via MLC1 and GAP45 to the rest of the IMC [22, 25]. Although such an orientation is not formally demonstrated for MyoH, it would be plausibly positioned like MyoA to interact with actin filaments underneath the plasma membrane.

MyoH is associated with MLC1, MLC5 and MLC7. Although MLC5 was previously shown to be at the conoid, localization of MLC7 was overlooked, likely due to the overexpression of a second copy driven by a constitutive promoter [39]. Intriguingly and quite unexpectedly, MLC5 and MCL7 are simultaneously dispensable and hence not absolutely required to sustain or regulate MyoH function. In contrast, MLC1, which is also shared by MyoA and MyoC [25], is not dispensable [24], hampering investigation of its role in MyoH function. MLC5 is exclusively associated with MyoH at the conoid since it is no longer detectable at this location upon depletion of MyoH. In contrast, MLC7 is still present at the conoid following MyoH depletion. In this context it is worth mentioning that other yet uncharacterized myosins could be present in the same location.

Conditional depletion of MyoH resulted in a block in motility, invasion and egress in tissue culture that translated into a complete avirulence in the murine infection model, indicating that the absence of MyoH is deleterious during the asexual part of the *T*. *gondii* life cycle. Further phenotypic investigations of *myoH-iKD* revealed that neither the secretion of the contents from the apical secretory organelles, nor protrusion of the conoid were impacted by the absence of MyoH. Taken together these results led us to conclude that a direct impairment of this actomyosin system accounts for the incapacity of the parasites to invade or egress. A closer examination of the fate of the CJ using anti-RON4 antibodies in parasites lacking either MyoH or MyoA was tremendously informative. In the absence of MyoA, the CJ is blocked at the beginning of the apical cap whereas in the absence of MyoH the CJ fails to form and progress, resulting in the presence of a dot at the extreme apical tip of the parasite probably blocked at the PCRs level. In light of these findings, we propose a model whereby MyoH acts as the initiator of gliding motility, potentially bringing the adhesin/receptor complexes to the cap where MyoA takes the relay and translocates the complexes to the posterior pole of the parasites (Fig 7B). Additionally the helical organization of the MTs of the conoid plausibly confers the helical movement to the parasites (Fig 7C), which is likely relayed at the level of the IMC by the array of inner membranous particles that are connected to the helical subpellicular MTs [44]. Finally, to explain the complete block in motility observed in absence of MyoH, it is tempting to speculate that this motor serves as a gatekeeper and allows polymerized actin to enter the glideosome space between the plasma membrane and the IMC.

Numerous questions still remain unanswered. Upon calcium stimulation the conoid protrudes, but in a MyoH-independent manner. The role of this protrusion and the motor generating it are unknown. A previous study reported the identification of an inhibitor (number 6) that affects conoid protrusion but not parasite motility, indicative of a role in host cell entry only [17].

Interestingly, a physical link between the APR and the rest of the conoid is established by RNG2, a protein recently shown to link the polar ring to the conoid [13]. Intriguingly, RNG2 localizes to the APR and flips when the conoid is protruded and could potentially ensure the relay between MyoH and MyoA. This hypothesis is supported by the phenotype observed in the *rng2-iKD* parasites, i.e. an impairment in gliding motility, which might not be solely explained by the reported decrease in microneme secretion [13]. AKMT is located at the conoid and its depletion displays a similar phenotype as the absence of MyoH [14]. Here we show that MyoH depletion does not prevent the cytosolic AKMT re-localization suggesting that the motor acts either independently or downstream of AKMT. Speculatively, AKMT could be involved in the methylation of the α-tubulin forming the conoid, given that *T*. *gondii* α-tubulin has been shown to be methylated [45]. In this proposed model MyoH, MyoA and MyoC participate in gliding motility that crucially depends on a physical connection between surface adhesins and the actomyosin system to generate power [46]. The central contribution of MyoH in motility and invasion reinforces the fundamental role of actin in establishment of infection. The connection between F-actin and the tail of the adhesins was thought to be mediated by aldolase, however recent studies ruled out a role of this glycolytic enzyme in gliding motility and invasion and hence the identification of a physiologically relevant connector is necessary to validate the overall model [47]. In addition to the coccidian subgroup of Apicomplexa, the Gregarines also harbour a closed conoid however we failed so far to identify a *MyoH* homologue in the available genome sequences of *Gregarina niphandrodes* (http://eupathdb.org). Moreover *MyoH* phylogeny based on the head domain showed a heterogeneous distribution within Apicomplexa with homologues also found in *Eimeria*, *Cryptosporidium* and Piroplasmida (*Babesia* and *Theileria*) [20]. It is unclear if these closely related motors preserved a similar function or evolved after divergence of these lineages. In this context it is worth mentioning that the invasive stages of the malaria parasites exhibit an electron dense zone at the tip of the zoite where the class XIV PfMyoB has recently been localized [48]. It is tempting to speculate that PfMyoB might fulfil at least in part a similar role as MyoH. PfMyoB is not anchored to MTs but is instead associated with an unusual light chain, which might bring this indispensable motor to its site of action, as reported for MyoA [22, 48].

## Materials and Methods

### Bacteria, parasite strains and culture

*E*.*coli* XL-10 Gold chemocompetent bacteria were used for all recombinant DNA experiments. *T*. *gondii* tachyzoites (RHΔ*hxgprt*, RHΔ*hxgprt*Δ*Ku80* and derivatives) were grown in human foreskin fibroblasts (HFF) or in Vero cells (African green monkey kidney cells) maintained in Dulbecco’s Modified Eagle’s Medium (DMEM, Gibco) supplemented with 5% foetal calf serum (FCS), 2 mM glutamine and 25 μg/ml gentamicin. Conditional expression of the different transgenes was performed with 0.5 μM Shld-1 for DD-fusion stabilization [38] and with 1 μg/ml anhydrotetracycline (ATc) for the Tet-inducible system [21].

### Cloning of DNA constructs

Genomic DNA was isolated with the Wizard SV genomic DNA purification system (Promega). RNA was isolated using Trizol (Invitrogen) extraction. Total cDNA was generated by RT-PCR using the Superscript II reverse transcriptase (Invitrogen) according to manufacturer’s protocol. TgMyoH GenBank accession number: XP_002366825. TgMyoH-NT and TgMyoH-T fragments were amplified on cDNA using primer pairs MyoH-3747-11F/MyoH-3749-12R and MyoH-3748-13F/MyoH-3749-12R (S1 Table), respectively and cloned into the pTub8DDmycFH2-HX [49] vector between *Nsi*I and *Pac*I sites. The GFP coding fragment was digested with *Nsi*I from the pT8DDmycGFPTgMyoF-tail-HX-Ble vector [50] and sub-cloned into the unique *Nsi*I site of DD-MyoH-NT to generate DD-GFP-TgMyoH-NT vector. TgMyoH-NTΔATS1 fragment was amplified from TgMyoH-NT using MyoH-5395-ΔATS1-18F/MyoH-5396-ΔATS1-19R primers following Q5 Site-Directed Mutagenesis Kit (NEB) manufacturer’s recommendations. To generate TgMyoH-3Ty, a gDNA fragment was amplified with primers MyoH-2748-1F/MyoH-2749-2R, digested with *Kpn*I and *Pst*I restriction enzymes and cloned into pT8-TgMIC13-3Ty-HX [51] between *Kpn*I and *Nsi*I sites. To generate 5’MyoH-TATi1-HX-tetOpS1MycNtMyoH vector, the 5’MyoH fragment was amplified using primers MyoH-3964-3F/MyoH-3965-4R and cloned between *Nco*I and *Bam*HI in 5’MyoF-TATi1-HX-tetO7S1MycNtMyoF [50]. NtMyoH was amplified with primers MyoH-3966-5F/MyoH-3967-6R and cloned between *Bgl*II and *Spe*I in the same plasmid. To generate TgMLC5-3Ty, TgMLC7-3Ty and MLC3-3Ty, gDNA fragments were amplified with primers MLC5-4129-19F/MLC5-1374-20R, MLC7-4403-24F/MLC7-2348-25R and MLC3-4402-31F/MLC3-1910-32R respectively and cloned into pT8-TgMIC13-3Ty-HX [51] between *Kpn*I and *Nsi*I sites. To generate MyoH-3Ty-LoxP-3’UTR-LoxP-U1, the C-terminal sequence from the TgMyoH-3Ty was digested with *Kpn*I and *Pst*I and sub-cloned into the *Kpn*I and *Nsi*I sites of pG152-3Ty-LoxP-3’UTRSag1-HXGPRT-LoxP-U1 [32] after modification to introduce a unique *Kpn*I site.

To generate vectors with gRNA specific of MLC5, MLC7, and MyoH, a directed mutagenesis was performed on the CRISPR/Cas9-GFP vectors [40] using the Q5 Site-Directed Mutagenesis Kit Protocol and the primer pairs gRNA-Rv-4883-33R/MLC5-gRNA-5087-21, gRNA-Rv-4883-33R/MLC7-gRNA-5068-26 and gRNA-Rv-4883-33R/MyoH-gRNA-5389-29 following the manufacturer recommendations.

pTub5-Cre vector [52] has been modified by insertion of a GFP coding sequence in frame with the Cre recombinase to create pTub5-Cre-GFP.

### Parasite transfection and selection of stable transfectants

*T*. *gondii* tachyzoites were transfected by electroporation as previously described [53]. *Δku80* [54, 55] strain was transfected with 40 μg of the plasmids: 5’MyoH-TATi1-HX-tetO7S1MycNtMyoH (linearized with *Sbf*I/*Spe*I) or TgMyoH-3Ty (linearized with *Xho*I) or MyoH-3Ty-LoxP-3’UTR-LoxPU1 (linearized with *Nsi*I). Stable transfectants were selected for hypoxanthine-xanthine-guanine-phosphoribosyltransferase (HXGPRT) expression in the presence of mycophenolic acid and xanthine as described earlier [56]. Parasites were cloned by limiting dilution in 96 well plates and analysed for the expression of the transgenes by indirect immunofluorescence assay (IFA).

### Transient transfection of fluorescent markers and Cre-GFP vectors

Transient transfection of CAM1-GFP and EGFP-TgCentrin2, provided by Ke Hu (Indiana University), was performed by using 40 μg of each plasmid as previously described [53]. 40 μg of the pTub5-Cre-GFP was transiently transfected in the Δ*ku80* and MyoH-3Ty-floxU1 strains 30 h and 48 h prior to performing egress and invasion assays, respectively.

### MLC5 and MLC7 knock out and MyoH knock in using CRISPR/Cas9

To disrupt *MLC5* and *MLC7* locus, 30 μg of the MLC5 gRNA-specific CRISPR/Cas9-GFP vector was transfected in RH parasites. After confirmation of gene disruptions, the MLC7 gRNA-specific CRISPR/Cas9-GFP vector was transfected in the *mlc5-KO* background or in wt RH to obtain respectively the *mlc5/7-KOs* and *mlc7-KO*. *TgMyoH* was subsequently endogenously tagged using 30 μg of the gRNA-specific CRISPR/Cas9-GFP co-transfected with the MyoH-Ki-2748-1F/ MyoH-Ki-RH-5390-30R PCR product amplified from the TgMyoH-3Ty vector. 48 h after transfections, parasites were FACS sorted and cloned into 96-well plates.

### Antibodies, indirect immunofluorescence and confocal microscopy

mAbs α-Ty tag BB2, α-MLC1 [57] and GFP-trap (Chromotek) antibodies were used to performed co-IPs. Antibodies described here were used for indirect immunofluorescence assay (IFA) and western blot analysis. The mAbs α-Ty tag BB2, α-Myc tag 9E10, α-SAG1 T4-1E5, α-MIC3 T42F3, α-ROP2 T3-4A7, as well as the polyclonal Abs αMIC4, α-PRF, α-GAP45, α-GAP40, α-CAT were previously described [22, 58–62]. Monoclonal α-RNG1 and polyclonal α-AKMT Ab were kindly provided by Dr. Naomi Morrissette (California University) and Dr. Ke Hu (Indiana University) respectively. α-tubulin antibody (#32–2500, Invitrogen) was used.

For western blot analysis, secondary peroxidase conjugated goat α-rabbit and α-mouse Abs (Molecular Probes, Paisley, UK) were used.

For IFAs, parasite-infected HFF cells seeded on cover slips were fixed with 4% paraformaldehyde (PFA) or 4% PFA/0.05% glutaraldehyde (PFA/GA) in PBS, depending of the antigen to be labelled. Fixed cells were then processed as previously described [63]. Confocal images were collected with a Zeiss microscope (LSM700, objective apochromat 63x /1.4 oil) at the Bioimaging core facility of the Faculty of Medicine, University of Geneva. Stacks of sections were processed with ImageJ and projected using the maximum projection tool.

### Western blot analysis of *T*. *gondii* tachyzoites

Crude extracts of *T*. *gondii* tachyzoites were subjected to SDS-PAGE. Western blot analysis was carried out using polyacrylamide gels under reducing conditions. Proteins were transferred to hybond ECL nitrocellulose. Primary and secondary antibodies are diluted in PBS, 0.05% Tween20, 5% skimmed milk. Bound antibodies were visualized using the ECL system (Amersham).

### Plaque assays

Cells were infected with about 50 parasites and let to develop for 7 days. Fixation and staining were performed as previously described [58].

### Intracellular growth assay

Similarly to plaque assays HFF were infected with parasites but fixation occurred after 24h with PFA/GA and stained with anti-TgGAP45. The number of parasites per vacuole was determined by counting the parasites in 100 vacuoles in duplicate for three independent experiments.

### Conoid protrusion assay

Freshly egressed *T*. *gondii* tachyzoites were pelleted and re-suspended in either: 20mM HEPES, 5 mM CaCl_2_ for calcium ionophore A23187 (3 μM) stimulation, or 20 mM HEPES, 138 mM NaCl, 2.7 mM KCl, 10% FCS, pH 7.2 for EtOH (2%) stimulation and same volume of DMSO or water was added as controls respectively. Parasites were then incubated for 8 min at 37°C on Poly-L-lysine coated coverslips and fixed for 30 min with PFA/GA. Protruded and non-protruded parasites were counted using the 63x magnification under DIC condition. The average number of protruded parasites was determined by counting 100 parasites for each condition for three independent experiments. For *Δku80* and *myoH-iKD*, parasites were pre-treated for 48 h ± ATc. Data are presented as mean values ± SD from three independent experiments. The significance of the data was evaluated using a parametric paired t-test.

### Invasion assay

The red/green assay was performed as previously described [64]. The number of intracellular and extracellular parasites was determined by counting 100 parasites in triplicate for three independent experiments. For *Δku80* and *myoH-iKD*, parasites were pre-treated for 48 h ± ATc.

### Attachment assay

Attachment of *T*. *gondii* parasites to HFF monolayers was assessed as previously described [65]. Briefly, extracellular wt expressing GFP and *myoH-iKD* cell lines pre-treated 48 h ± ATc were mixed 50/50 in Endo buffer (44.7 mM K2SO4, 10 mM Mg2SO4, 106 mM sucrose, 5 mM glucose, 20 mM Tris, 0.35% wt/vol BSA, pH 8.2) containing 1 μM cytochalasin D (Sigma-Aldrich). Incubate at room temperature for 10 min. 500 μL was added to a coverslip coated with an HFF monolayer (assay) or Poly-L-lysine (control) and centrifuged for 1 min at 1000 rpm. Controls samples were fixed with PFA/GA for 7 min at RT. Then, medium was replace with pre-warmed DMEM 5% FCS containing 1 μM cytochalasin D to prevent invasion, incubated for 15 min at 37°C and fixed with PFA/GA for 7 min. Immunofluorescence was performed using α-GAP45 and Alexa647 (“red”) as secondary antibodies. Ratio between red/green (wt) and red only (*myoH-iKD*) attached parasites was counted in triplicate (minimum of 100 parasites screened) for three independent experiments either for controls or assays.

### E-vacuole assays

E-vacuole assays utilizing cytochalasin D were performed as described [65].

### Pulse invasion assay

Freshly released parasites were inoculated on new host cells, centrifuged for 1 min at 1000 g and allowed to invade for 7 minutes before fixation with PFA/GA for 5 minutes. IFAs were performed first without permeabilization with α-SAG1 antibodies then fixed 10 min with 1% formaldehyde and permeabilized with 0.1% Saponin/PBS for 20 min at RT and stained with α-RON4. Only parasites harbouring a RON4 positive signal were counted. Ratio between the different conformations (attached—rhoptry content secreted, conoid invaded or post conoid invaded) were counted in triplicate (minimum of 100 parasites screened) for three independent experiments. Data are presented as mean values ± SD from three independent experiments.

### Egress assay

Δ*ku80* and *myoH-iKD* were grown for 18 h ±ATc. Freshly egressed tachyzoites were added to a new monolayer of HFF and grown for 30 h ±ATc. The infected HFF were then incubated for 5 min at 37°C with DMEM containing either 3 μM of the Ca^2+^ ionophore A23187 (from *Streptomyces chartreusensis*, Calbiochem) or DMSO as a negative control. Host cells were fixed with PFA/GA and IFA were performed using α-GAP45 antibodies. The average number of egressed vacuoles was determined by counting 200 vacuoles per strain and per condition. Data are presented as mean values ± SD from three independent experiments. The significance of the data was evaluated using a parametric paired t-test and the two-tailed p-value is written on the corresponding graph.

### Subcellular fractionation of *T*. *gondii*

Freshly egressed tachyzoites were harvested, washed in PBS and then re-suspended into PBS, PBS/ 1% Triton-X-100, PBS/ 1 M NaCl, or PBS/ 0.1 M Na2CO3, pH 11.5. Parasites were lysed by freezing and thawing followed by sonication on ice. Pellet and soluble fractions were separated by centrifugation at 14 000 rpm for 1 h at 4°C.

### Metabolic labelling and co-immunoprecipitation (co-IP)

Metabolic labelling of the tachyzoites was done with 50 mCi [35S]-labeled methionine/cysteine (Hartmann analytic GmbH) per ml for 4 h at 37°C followed by co-IP as previously described [25].

### Electron microscopy

Infected host cells were washed with 0.1 M phosphate buffer pH 7.4 and were fixed with 2.5% glutaraldehyde in 0.1 M phosphate buffer pH 7.4, post-fixed in osmium tetroxide, dehydrated in ethanol and treated with propylene oxide prior to embedding in Spurr’s epoxy resin. Thin sections were stained with uranyl acetate and lead citrate prior to examination using a Technai 20 electron microscope (FEI Company). Samples for EM were prepared twice independently and multiple thin sections for each sample were examined.

### Gliding assay

Trail deposition assays have been performed as described before [6]. Briefly, freshly released parasites were allowed to glide on Poly-L-Lysine-coated glass slides for 15 min at 37°C before they were fixed with PFA/GA and stained with SAG1. *myoH-iKD* parasites were treated for 48 h ± ATc.

The percentage of produced trails was determined by counting the number of anti-SAG1 labelled trails for 1000 parasites. Data are presented as percentage mean values ± SD from three independent experiments. The significance of the data was evaluated using a parametric paired t-test and the two-tailed p-value is written on the corresponding graph.

### Deoxycholate extraction

Freshly egressed parasites were attached to Poly-L-Lysine-coated coverslips and either treated with 10 mM deoxycholate for 10 min at room temperature. Parasites were then fixed with PFA/GA for 10 min and then proceeded as for IFAs. DD-MyoH-NT parasites were pre-treated for 24 h with Shield-1.

### Microneme secretion assay

*T*. *gondii* tachyzoites freshly egressed from host cells were harvested by centrifugation at 240 g, RT for 10 min and the pellet washed twice in intracellular buffer (5 mM NaCl, 142 mM KCl, 1 mM MgCl2, 2mM EGTA, 5.6 mM glucose and 25 mM HEPES, pH 7.2) prewarmed to 37°C. Control parasites were treated 30 min at room temperature with 1 μM of compound 2 [34, 35, 66] to prevent microneme secretion. Pellets were resuspended in egress buffer (141.8 mM NaCl, 5.8 mM KCl, 1 mM MgCl2, 1mM CaCl2, 5.6 mM glucose and 25 mM HEPES, pH 7.2) ± ethanol (2%), incubated 30 min at 37°C. Parasites were centrifuged at 1000g for 5 min, 4°C. Pellets were washed once in PBS and stored before Western blot. Supernatants were centrifuged at 2000 g for 5 min, 4°C and supernatant was used as ESA (excreted/secreted antigen). Pellets and ESA samples were analysed for AMA1, MIC2, MIC6, MIC4, Myc, catalase and dense granule (GRA1) by Western blot. For *Δku80* and *myoh-iKD*, parasites were pre-treated for 48 h ± ATc.

## Supporting Information

S1 Fig Alignment of Coccidian Myosin H. Muscle [67] multiple alignment of the *T*. *gondii*, *Neospora caninum*, *Sarcocystis neurona*, *Eimeria tenella* MyoH sequences. Identical residues are in red (51%), strongly similar residues in green (17%) and weakly similar residues in blue (7.5%). Accession numbers from EupathDB [68]: TgMyoH (TGME49_243250), NcMyoH (NCLIV_018020), SnMyoH (TC5K_7772_1) and EtMyoH (ETH_00017965). The head domain is highlighted in yellow, the IQ motifs in green and the RCC1/ATS1 domains in blue.
(TIF)

S2 Fig MyoH knock-out strategy and phenotypes. (A) Strategy used to generate the *myoH-iKD* parasite line. (B) PCR analyses performed on gDNA assessed the correct integration of the transfected construct. Expected sizes of the PCR products are indicated on the scheme and the sequence of the primers are listed in S1 Table. (C) A severe defect in the lytic cycle was observed when MyoH was depleted by ATc as observed by plaque assay fixed 7 days after inoculation. (D) IFAs of microneme (MIC3) and rhoptry (ROP2) organelles upon MyoH depletion for 48 h revealed no apparent defect in organelle morphology. Scale bar 2 μm. (E) IFAs representative of the parasite pools obtained 30 h after transient transfection of Cre-GFP in the MyoH-3Ty-floxU1 strain. Cre-GFP negative vacuoles (GFP negative) showed no excision with the concomitant presence of MyoH-3Ty (upper panel). Cre-GFP positives vacuole (GFP positive) showed no signal for MyoH-3Ty confirming its down regulation. Scale bar 2 μm. (F) Calcium ionophore-induced egress assay of MyoH-3Ty-floxU1 and parental (*Δku80*) strains performed 30 h after transfection of Cre-GFP expressing vector by treating the parasites with A23187 for 7 min. Only parasites expressing Cre (GFP positive) were taken into account for the quantification. Results are expressed as percentage of GFP positive ruptured vacuoles and represented as mean ± SD. (G) The invasion capacity of MyoH-3Ty-flox-U1 and parental (*Δku80*) parasites was evaluated 30 h after Cre-GFP transfection. Results are expressed as percentage of Cre expressing invading parasites (GFP positive) and represented as mean ± SD. (I) IFAs of MIC2 and SAG1 proteins on non-permeabilized extracellular parasites stimulated by A23187. No difference was observed regarding the MIC2 staining at the parasite surface in presence or absence of MyoH. Scale bar 2 μm. (J) MLC1 staining in *myoH-iKD* ± ATc. No apparent defect in MLC1 localization at the IMC was observed. Scale bar 1 μm. (H) Microneme secretion assay performed on wt (*Δku80*) and *myoH-iKO* lines ± ATc for 48 h and analyzed by western blot using anti-MIC2 antibodies on parasites pretreated with compound 2. No secretion was observed. Catalase (CAT) was used as cytosolic control and dense granule 1 (GRA1) as control for constitutive secretion. ESA: excreted/secreted antigens, ESA-induced: induction with 0.5 M ethanol. For (F) and (G), the significance of the results was assessed using a parametric paired t-test and the two-tailed p-values are presented on the graphs.
(TIF)

S3 Fig *In vivo* characterization of *myoH-iKD* and interactions with myosin light chains. (A) CD1 mice were infected with 15 tachyzoites of RH (in black) or *myoH-iKD* ± ATc (respectively in red and blue) strains and monitored over 20 days. A challenge with ∼1000 wild-type *Δku80* tachyzoites was performed on mice that survived initial infection. Mice were monitored for 10 more days and five mice were infected per condition. (B) Western blot analysis of the seroconversion of mice used in the virulence assay described in panel A. *mic8-iKD* [69] was used as an avirulent (-ATc) and avirulent/non-seroconverting (+ATc) control. (C) Western blot analysis using anti-Myc antibodies shows stabilization of DD-Myc-GFP-MyoH-NT (126 kDa) after 24 h of Shield-1 (Shld-1) treatment. GAP40 serves as loading control. (D) DD-Myc-GFP-MyoH-NT localize to the conoid after 24 h of Shield-1 (Shld-1). (E) SDS PAGE gel of co-IP experiments performed with GFP-Trap beads on DD-Myc-GFP (control) and DD-Myc-GFP-MyoH-NT strains and metabolically labeled with [S^35^]-methionine/cysteine. No band corresponding to the MyoH wt (170 kDa) was detected suggesting no heterodimer (MyoH-wt&DD-Myc-GFP-MyoH-NT) formation. (F) Conditional stabilization of DD-MyoH-NT after 24 h Shld-1 resulted with an abnormal apicoplast (Cpn60) inheritance and mislocalization of rhoptries (ROP2) but no difference for micronemes localization (MIC3). In sharp contrast, the *myoH-iKD* strains treated with ATc for 48 h showed no phenotype for the localization of these three organelles. (G) TgMLC3-3Ty is found at the predicted molecular weight by western blot (106 kDa). GAP40 was used as loading control. (H) Silver stained SDS PAGE gel of co-IP experiments performed with anti-Ty antibodies on MLC5-3Ty and MLC7-3Ty strains. Asterisks correspond to heavy and light chains of the anti-Ty antibodies. Bands corresponding to the size of MyoH (zones A1 and B1) were cut and sent for mass spectrometry. (I) Peptides identified are indicated in yellow on the MyoH sequence and the raw data of peptides obtained after mass spectrometry are listed in the S1 Dataset.
(TIF)

S4 Fig MyoH is associated with MLC1 independently of MLC5 and MLC7. (A) Genomic sequences comparison between *MLC5* or *MLC7* wt and *mlc5-KO*, *mlc5&7-KOs and mlc7-KO* parasite lines. CRISPR/Cas9 mediated cleavage resulted with frameshifts in the coding sequences producing premature stop codons (in red). gRNA sequences are highlighted in green. (B) Co-IP experiments performed with anti-MLC1 antibodies to detect MyoH-3Ty in wt and in *mlc5&7-KO* strains and followed by western blot analyses. MyoH was detected, demonstrating its association with MLC1 in wt and *mlc5&7-KO*. MyoA was used as positive control to confirm the glideosome components precipitation. Profilin (PRF) and α-tubulin (TUB) were used as negative controls. (C) IFAs of MLC1 and MyoH-3Ty (arrowhead) on extracellular parasites treated with 3 μM of A23187. MLC1 localization is not altered by *mlc*5&7 genes deletion.
(TIF)

S1 Table Oligonucleotides primers used in this study. Oligonucleotides primers used in this study. Letters in brackets correspond to primers of S2 Fig. F: forward, R: reverse.
(DOCX)

S1 Movie Three-dimensional reconstruction of the MyoH-3Ty staining by super-resolution microscopy (SIM) MyoH-3Ty is in green and IMC1 is shown in red. (MOV)

S2 Movie Induced egress assay of *myoH-iKD* parasites without ATc treatment recorded at two frames per second and played at 50 frames per second. Time is indicated in minutes:seconds and scale bar represent 10 μm.
(MOV)

S3 Movie Induced egress assay of *myoH-iKD* parasites after 48 h of ATc treatment recorded at two frames per second and played at 50 frames per second. Time is indicated in minutes:seconds and scale bar represent 10 μm.
(MOV)

S1 Dataset Raw data from the MS analysis of zones A1 and B1 (S3H Fig): overview of percentage of spectra, protein probabilities, spectrum counts and unique spectrum counts. (XLSX)
