# Supplementary figures and images for: The Conoid Associated Motor MyoH Is Indispensable for Toxoplasma gondii Entry and Exit from Host Cells
Source: PLoS Pathog. 2016 Jan 13;12(1):e1005388. doi: 10.1371/journal.ppat.1005388 (PMC4711953; doi:10.1371/journal.ppat.1005388)

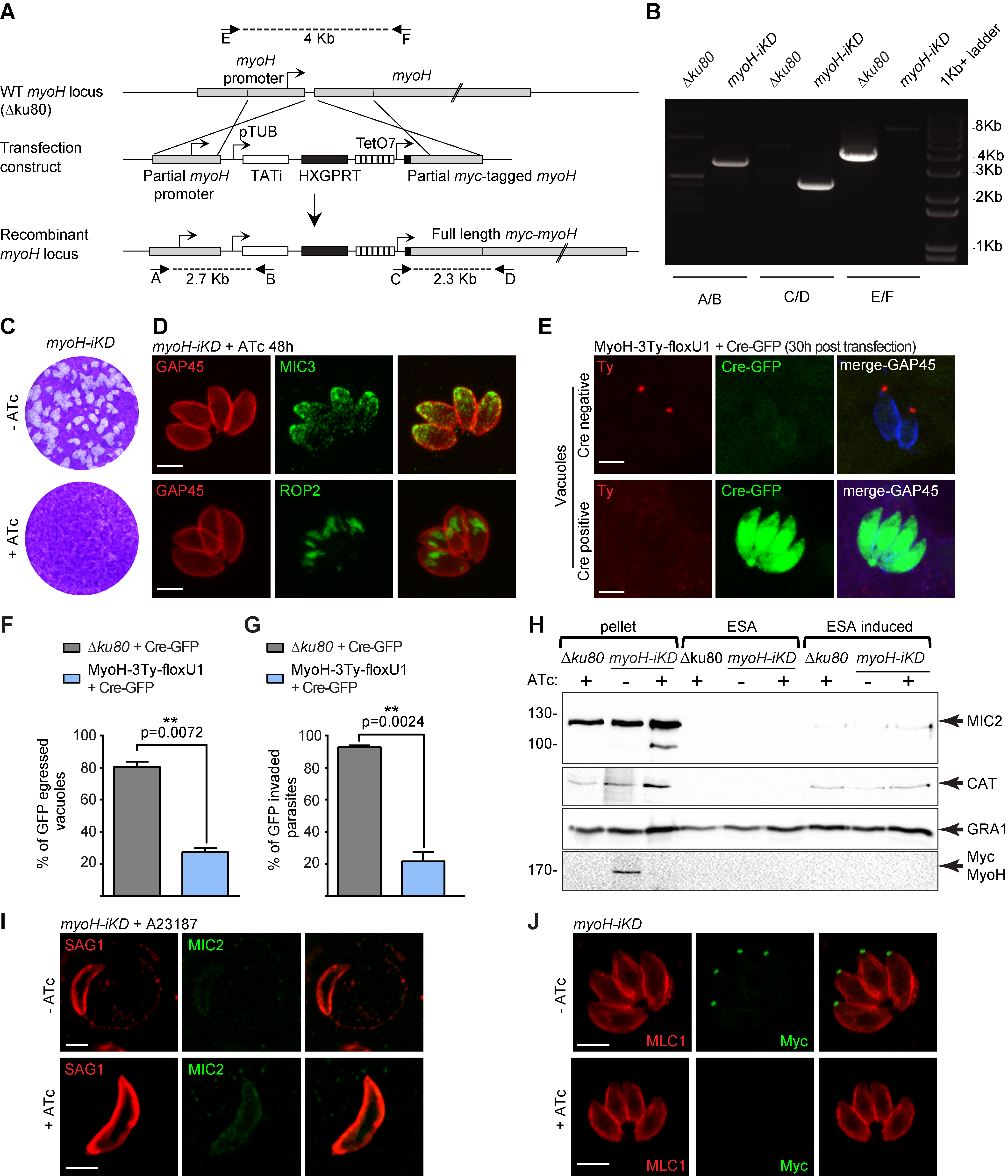

Supplement: S2 Fig — (A) Strategy used to generate the myoH-iKD parasite line. (B) PCR analyses performed on gDNA assessed the correct integration of the transfected construct. Expected sizes of the PCR products are indicated on the scheme and the sequence of the primers are listed in S1 Table. (C) A severe defect in the lytic cycle was observed when MyoH was depleted by ATc as observed by plaque assay fixed 7 days after inoculation. (D) IFAs of microneme (MIC3) and rhoptry (ROP2) organelles upon MyoH depletion for 48 h revealed no apparent defect in organelle morphology. Scale bar 2 μm. (E) IFAs representative of the parasite pools obtained 30 h after transient transfection of Cre-GFP in the MyoH-3Ty-floxU1 strain. Cre-GFP negative vacuoles (GFP negative) showed no excision with the concomitant presence of MyoH-3Ty (upper panel). Cre-GFP positives vacuole (GFP positive) showed no signal for MyoH-3Ty confirming its down regulation. Scale bar 2 μm. (F) Calcium ionophore-induced egress assay of MyoH-3Ty-floxU1 and parental (Δku80) strains performed 30 h after transfection of Cre-GFP expressing vector by treating the parasites with A23187 for 7 min. Only parasites expressing Cre (GFP positive) were taken into account for the quantification. Results are expressed as percentage of GFP positive ruptured vacuoles and represented as mean ± SD. (G) The invasion capacity of MyoH-3Ty-flox-U1 and parental (Δku80) parasites was evaluated 30 h after Cre-GFP transfection. Results are expressed as percentage of Cre expressing invading parasites (GFP positive) and represented as mean ± SD. (I) IFAs of MIC2 and SAG1 proteins on non-permeabilized extracellular parasites stimulated by A23187. No difference was observed regarding the MIC2 staining at the parasite surface in presence or absence of MyoH. Scale bar 2 μm. (J) MLC1 staining in myoH-iKD ± ATc. No apparent defect in MLC1 localization at the IMC was observed. Scale bar 1 μm. (H) Microneme secretion assay performed on wt (Δku80) and myoH-iKO lines ± [file ppat.1005388.s002.tif]

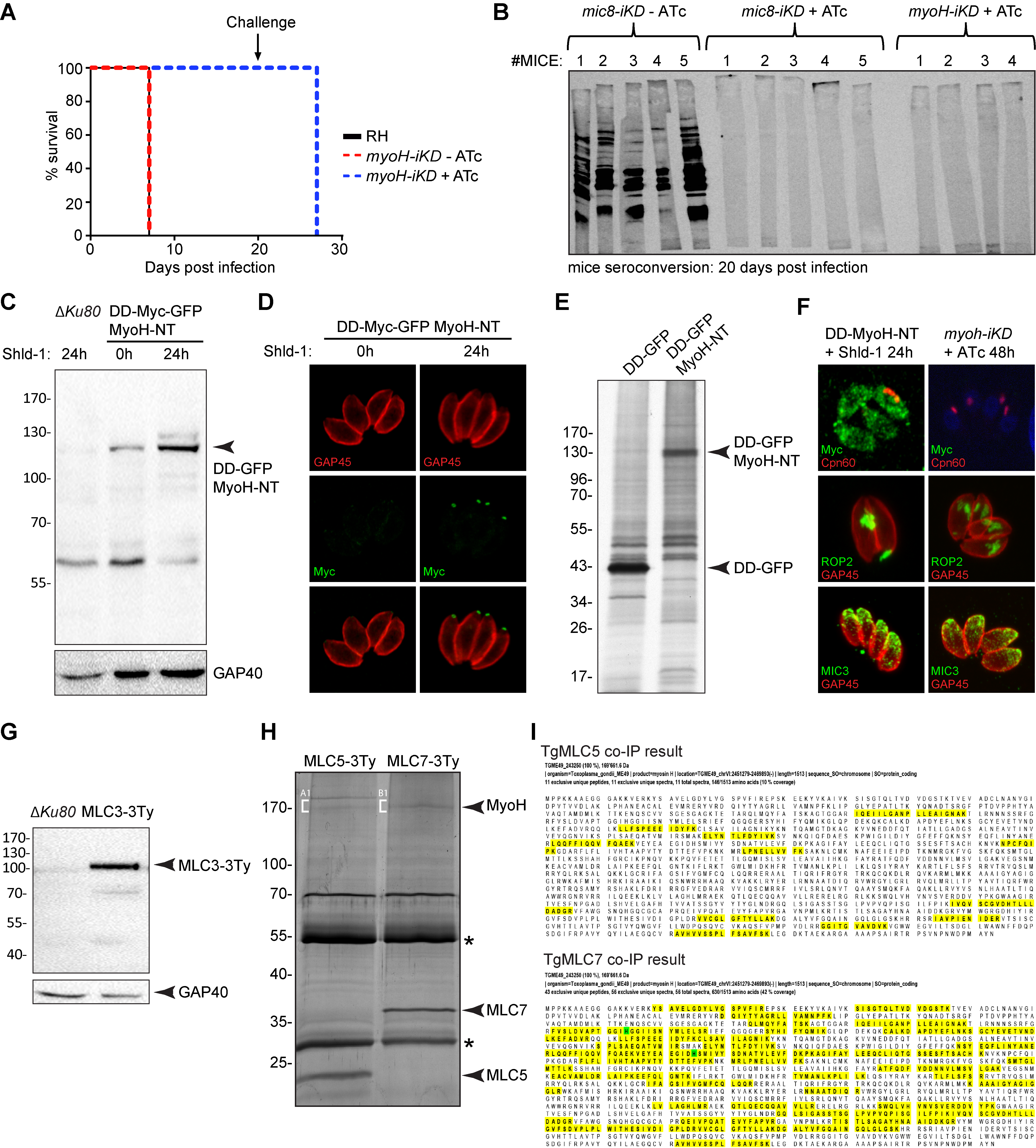

Supplement: S3 Fig — (A) CD1 mice were infected with 15 tachyzoites of RH (in black) or myoH-iKD ± ATc (respectively in red and blue) strains and monitored over 20 days. A challenge with ∼1000 wild-type Δku80 tachyzoites was performed on mice that survived initial infection. Mice were monitored for 10 more days and five mice were infected per condition. (B) Western blot analysis of the seroconversion of mice used in the virulence assay described in panel A. mic8-iKD [69] was used as an avirulent (-ATc) and avirulent/non-seroconverting (+ATc) control. (C) Western blot analysis using anti-Myc antibodies shows stabilization of DD-Myc-GFP-MyoH-NT (126 kDa) after 24 h of Shield-1 (Shld-1) treatment. GAP40 serves as loading control. (D) DD-Myc-GFP-MyoH-NT localize to the conoid after 24 h of Shield-1 (Shld-1). (E) SDS PAGE gel of co-IP experiments performed with GFP-Trap beads on DD-Myc-GFP (control) and DD-Myc-GFP-MyoH-NT strains and metabolically labeled with [S35]-methionine/cysteine. No band corresponding to the MyoH wt (170 kDa) was detected suggesting no heterodimer (MyoH-wt&DD-Myc-GFP-MyoH-NT) formation. (F) Conditional stabilization of DD-MyoH-NT after 24 h Shld-1 resulted with an abnormal apicoplast (Cpn60) inheritance and mislocalization of rhoptries (ROP2) but no difference for micronemes localization (MIC3). In sharp contrast, the myoH-iKD strains treated with ATc for 48 h showed no phenotype for the localization of these three organelles. (G) TgMLC3-3Ty is found at the predicted molecular weight by western blot (106 kDa). GAP40 was used as loading control. (H) Silver stained SDS PAGE gel of co-IP experiments performed with anti-Ty antibodies on MLC5-3Ty and MLC7-3Ty strains. Asterisks correspond to heavy and light chains of the anti-Ty antibodies. Bands corresponding to the size of MyoH (zones A1 and B1) were cut and sent for mass spectrometry. (I) Peptides identified are indicated in yellow on the MyoH sequence and the raw data of peptides obtained after mass spectrometry are li [file ppat.1005388.s003.tif]

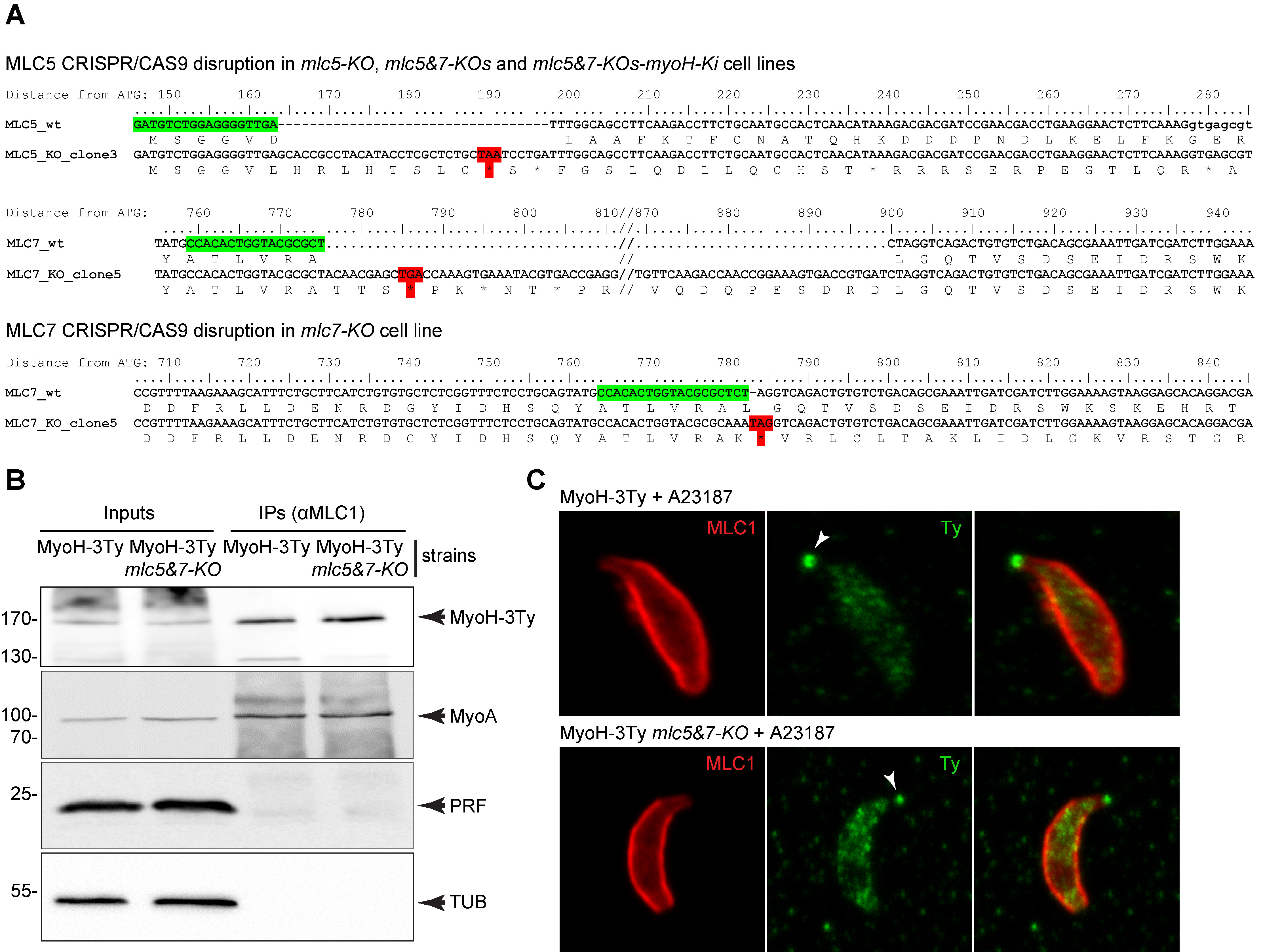

Supplement: S4 Fig — (A) Genomic sequences comparison between MLC5 or MLC7 wt and mlc5-KO, mlc5&7-KOs and mlc7-KO parasite lines. CRISPR/Cas9 mediated cleavage resulted with frameshifts in the coding sequences producing premature stop codons (in red). gRNA sequences are highlighted in green. (B) Co-IP experiments performed with anti-MLC1 antibodies to detect MyoH-3Ty in wt and in mlc5&7-KO strains and followed by western blot analyses. MyoH was detected, demonstrating its association with MLC1 in wt and mlc5&7-KO. MyoA was used as positive control to confirm the glideosome components precipitation. Profilin (PRF) and α-tubulin (TUB) were used as negative controls. (C) IFAs of MLC1 and MyoH-3Ty (arrowhead) on extracellular parasites treated with 3 μM of A23187. MLC1 localization is not altered by mlc5&7 genes deletion. (TIF) [file ppat.1005388.s004.tif]
