# Supplementary material for: The Conoid Associated Motor MyoH Is Indispensable for Toxoplasma gondii Entry and Exit from Host Cells
Source: PLoS Pathog. 2016 Jan 13;12(1):e1005388. doi: 10.1371/journal.ppat.1005388 (PMC4711953; doi:10.1371/journal.ppat.1005388)
Supplement: S1 Table — Oligonucleotides primers used in this study. Letters in brackets correspond to primers of S2 Fig. F: forward, R: reverse. (DOCX) [file ppat.1005388.s005.docx]

| Name | Sequence 5’-3’ |
| --- | --- |
| MyoH-Ki-2748-1F | CCGGGTACCTTGTGTATTTTTGATGCCCGCTCCCG |
| MyoH-Ki-2749-2R | GGCCTGCAGCGTTGTAGGCCATGGGATCCCAGTTCG |
| MyoH-iKD-5’-3964-3F (E) | CGCCCATGGCCTGCAGGCATCTGTTATGGATCACCACTGGATAC |
| MyoH-iKD-5’-3965-4R | CGCGGATCCGGGGGATTTCAAGGGGATTCGGAGCAG |
| MyoH-iKD-CDS-3966-5F | CGCGGATCCATGCCGCCAAAGAAGGCTGCCG |
| MyoH-iKD-CDS-3967-6R | CGCACTAGTGGCTTCGCGACTGGTGTGGAGAAG |
| MyoH-iKD-screen-4076-7F (A) | GCGAGGGCATTTCCGGAGGCTTTCTAA |
| MyoH-iKD-screen-4077-8R | ATCGGTTCTCGGTCTATGCTGCTCGTATT |
| MyoH-iKD-screen-4078-9F | CCGTGCTCTGCGTGGACCCTAAA |
| MyoH-iKD-screen-4092-10R (D) | CGAGAAAGTCTCCAGCGAGTGGC |
| MyoH-3747-11F | GATGCATATCGACAAACATTTCAGAACTGTCATGGC |
| MyoH-3749-12R | GTTAATTAATTAGTTGTAGGCCATGGGATCCC |
| MyoH-3748-13F | GATGCATCTGAAGTTGGTCCTTGCGGGTC |
| MyoH-2903-14R (B) | GAGCGAGTTTCCTTGTCGTCAGGCC |
| MyoH-1935-15F (C) | CGCTGCACCACTTCATTATTTCTTCTGG |
| MyoH-3964-16R | CGCCCATGGCCTGCAGGCATCTGTTATGGATCACCACTGGATAC |
| MyoH-4026-17F (F) | CAATCGCGAGGGAGAGTGAAATGGTTAC |
| MyoH-5395-ΔATS1-18F | GTCTACCAGTGGGGTGTCTTCCT |
| MyoH-5396-ΔATS1-19R | GCTCGTCGCCACGACTGTATG |
| MLC5-4129-19F | CGGGTTAACCTCGGCTTTTAGTGACCGTCTTC |
| MLC5-1374-20R | CCGATGCATTGGATTGGATAATGGCCGCCATCAT |
| MLC5-gRNA-5087-21 | GATGTCTGGAGGGGTTGATTGTTTTAGAGCTAGAAATAGC |
| MLC5-seqcing-3410-22F | GCGAGATCTTCGAGTGACGCGGATATTCAAATGGC |
| MLC5-seqcing-3411-23R | CGCACTAGTCTGTGCAGACAAGCAAGACACAC |
| MLC7-4403-24F | GGGGTACCGCCGGACTACCGTGCGCGT |
| MLC7-2348-25R | CCATGCATTAGTTGCATTGACTGTTGCGACTCCAGC |
| MLC7-gRNA-5068-26 | GCCACACTGGTACGCGCTCTGTTTTAGAGCTAGAAATAGC |
| MLC7-seqcing-5249-27F | GACGACTTTCGTCTTCTTGACGAG |
| MLC7-seqcing-5250-28R | CGGTAGTCCGGCGGCAGGTC |
| MyoH-gRNA-5389-29 | GCCTACAACTAAGATCTTTTGTTTTAGAGCTAGAAATAGC |
| MyoH-Ki-RH-5390-30R | GAAATTCGTAGGATTCTGGCCACCTGTCGCCAGGAAACAG  CTATGACCATG |
| MLC3-4402-31F | GGGGTACCAGCGTTGTGCGTATCGTCGAG |
| MLC3-1910-32R | CCGTTAATTAATCACTTCTGTGGGACGAGGGCGACGAGC |
| gRNA-Rv-4883-33R | AACTTGACATCCCCATTTAC |
